# Understanding Ion Transport in Alkyl Dicarbonates: An Experimental and Computational Study

**DOI:** 10.1021/acsphyschemau.4c00078

**Published:** 2024-11-10

**Authors:** Samuel Emilsson, Marcelo Albuquerque, Pernilla Öberg, Daniel Brandell, Mats Johansson

**Affiliations:** † Department of Fibre and Polymer Technology, Division of Coating Technology, KTH Royal Institute of Technology, SE-100 44 Stockholm, Sweden; ‡ Institute of Physics, Universidade Federal Fluminense (UFF), Praia Vermelha Campus, Boa Viagem, 24210-346 Niterói, Rio de Janeiro, Brazil; § Department of Chemistry—Ångström Laboratory, Uppsala University, Box 538, SE-751 21 Uppsala, Sweden

**Keywords:** alkyl dicarbonates, electrolyte, DMC, DEC, lithium ion, coordination number, end groups

## Abstract

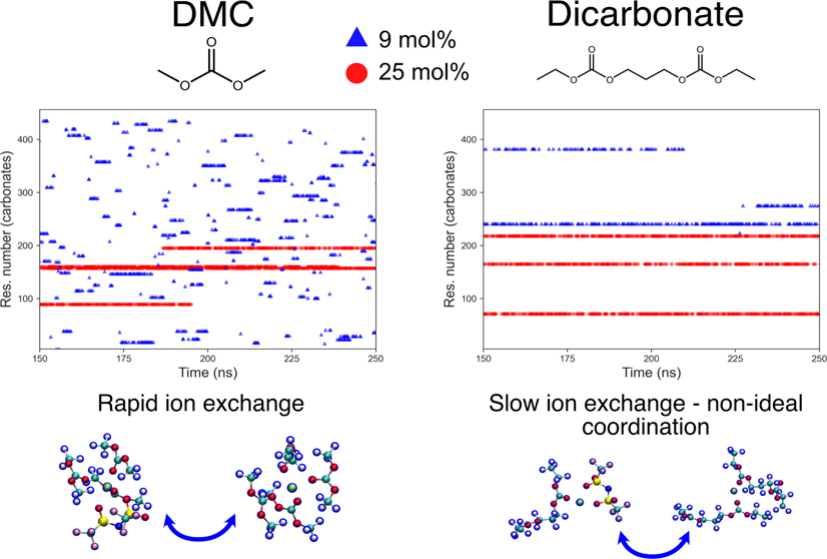

In an effort to improve safety and cycling stability of liquid electrolytes, the use of dicarbonates has been explored. In this study, four dicarbonate structures with varying end groups and spacers are investigated. The effect of these structural differences on the physical and ion transport properties is elucidated, showing that the end group has a significant influence on ion transport. The solvation structure and ion transport in the dicarbonates are compared to those of the linear carbonates dimethyl carbonate (DMC) and diethyl carbonate (DEC). Although the carbonate coordination numbers (CN) are similar in the different systems, the CN from the anion is higher in dicarbonate electrolytes. At low salt concentrations, rapid solvent exchange is observed in the DMC- and DEC-containing systems, transitioning to a more correlated ion transport at high salt concentration. In contrast, the exchange of solvents around lithium ions (Li^+^) is limited in the dicarbonate systems regardless of the salt concentration, with only one carbonate group from each molecule participating in the coordination. In addition, according to the molecular dynamics simulations, Li^+^ mainly moves together with coordinating dicarbonate molecules and anion(s).

## Introduction

The current state-of-the-art Li-ion battery electrolyte typically consists of lithium hexafluorophosphate (LiPF_6_) dissolved in a mixture of linear carbonates (DMC, DEC, and EMC) and ethylene carbonate (EC). This electrolyte mixture exhibits high ionic conductivity (10 mS cm^–1^) and has proven successful in forming stable solid-electrolyte interphase (SEI) on graphite^1^ and provides excellent cycling stability at ambient temperature, especially with the addition of film-forming additives like vinyl carbonate (VC) and ethylene sulfate (DTD).^2^

These carbonate-based liquid electrolytes have some limitations, however, which have led to extensive research into alternative electrolyte systems. First, the electrolytes are sensitive to higher temperatures, showing an Arrhenius-like rise in capacity fade and degradation.^3,4^ Furthermore, the high volatility of particularly the linear carbonates could pose a safety risk at elevated temperatures.^5^ Ultimately, this necessitates the use of extensive cooling systems in several battery applications, such as electric vehicles, lowering their driving range.^6,7^ Another limitation of these electrolytes is their lack of stability toward lithium metal (Li), due to inefficient plating and the formation of unstable SEI layers which lead to low Coulombic efficiencies (CE %) and rapid capacity fade.^8^

To counteract these two challenges, a wide variety of novel electrolyte technologies are being researched. For example, highly concentrated electrolytes (HCE)^9^ and localized highly concentrated electrolytes (LHCE)^10^ are studied as alternative liquid electrolytes. Going beyond liquid electrolytes, solid state electrolytes^11^ and solid polymer electrolyte^12,13^ are also heavily researched as alternatives. All of these technologies come with a set of benefits and challenges.^14^

Efforts have also increasingly circled back to moderately concentrated liquid electrolytes (LE), with the focus on developing novel solvent structures. A major contribution to this has been the better understanding of the solvation structure and its importance on (de)solvation and cycling stability.^15,16^ In general, a number of solvent molecules form a solvation shell around lithium ions (Li^+^), separating them from the anion. However, the solvation structure can be tuned by changing concentration (in HCE and LHCE)^10^ and by using additives or multiple salts,^17^ but also by changing the solvent itself. It is believed that if the solvent-Li^+^ interaction is weaker, there is a lower risk for solvent co-intercalation into graphite.^15^ Furthermore, the anion is usually present in the solvation shell in such cases and participates in the formation of the SEI layer, which can be beneficial for its stability.^18,19^ Several studies have focused on altering the structure of cyclic and linear ethers to achieve weaker solvation ability at moderate salt concentrations.^20−22^ All of these studies have mainly focused on how these alterations can increase cycling stability in high-voltage lithium metal batteries (LMB), but undoubtedly these changes in solvation structure also affect the ion transport in the electrolyte.

Recently, the use of dicarbonates (particularly dimethyl-2,5-dioxahexane carboxylate, DMOHC) as primary electrolyte solvent has been investigated.^23,24^ DMOHC and other dicarbonates have been known to provide degradation product in commercial carbonate electrolytes for a long time.^25^ They have often been considered problematic due to their high viscosity and low ionic conductivity. Hofmann et al. were the first to report the use of DMOHC as an additive to improve the cycling performance of LNMO cells at elevated temperatures.^26^ Dahn and colleagues investigated electrolytes with purely DMOHC or diethyl-2,5-dioxahexane carboxylate (DEOHC) together with either LiPF_6_ or LiFSI. Lithium ion cells were cycled at high temperatures, 70 and 85 °C, showing excellent stability.^3,23^ Cells containing DMOHC and LiFSI with NMC electrodes operated to 3.8 V have lifetimes of over 1 year at 85 °C with minimal gas generation and resistance growth. The thermal stability and formation of an apparently stable SEI were credited for the stable cycling performance. Zhang et al. further extended the use of dicarbonates by presenting the use of 1 M LiPF_6_ in DMOHC for high-voltage LMBs cycled at ambient temperatures. This work suggested that the improved cycling stability of the dicarbonate electrolyte was related to the weak Li^+^ interaction and substantial prevalence of the anion in the first solvation shell, which lead to a stable and anion-rich SEI and cathode-electrolyte interphase (CEI).^24^ These studies show some of the promising characteristics of dicarbonates but have focused little on understanding the ion transport in these electrolytes.

In this contribution, four dicarbonate structures are investigated with varying end groups and spacer lengths. The dicarbonates are combined with LiTFSI as a salt with increased thermal stability.^27^ The influence of these slight structural differences on the Li^+^ transport is systematically investigated. When comparing the viscosity and ionic conductivity, the dicarbonates clearly fall into two distinct groups, with the end group playing a more decisive role than the spacer length between the carbonate groups. The origin of these differences was investigated by examining the coordination environment with spectroscopic and computational techniques. In turn, molecular dynamics (MD) simulations were used to further examine the solvation structure and ion transport mechanism in dicarbonates compared to DMC and DEC.

## Experimental Section

### Materials

1,3-Propanediol (98%), ethylene glycol (99.8%), ethyl chloroformate (99%), and methyl chloroformate (99%) were purchased from Sigma-Aldrich. Pyridine (99.9%) was purchased from VWR. Molecular sieves (4 Å) and lithium bis(trifluoromethanesulfonyl)imide (LiTFSI) (99.99%) were purchased from Sigma-Aldrich.

### Synthesis

The four dicarbonates were synthesized by acylation of diols in the following procedure. 3 g of diol (1,3-propanediol for PE and PM or ethylene glycol for EE and EM) was dissolved in 100 mL of dichloromethane (DCM). The solution was cooled in an ice bath, and chloroformate (methyl chloroformate for PM and EM and ethyl chloroformate for PE and EE) was introduced dropwise using a dropping funnel to the reaction vessel in 2.2 or 2.5 molar ratios (for ethyl and methyl chloroformate, respectively) under stirring. Pyridine was subsequently added, also via a dropping funnel, at a 2.0 molar ratio relative to the chloroformate reagent. The reaction was left to proceed overnight without supplying additional ice and subsequently quenched with *N*,*N*-dimethylethanolamine. To purify the dicarbonates, the reaction mixture was extracted with 2 × 100 mL of deionized water and brine, 2 × 100 mL of 1 M HCl, and finally 100 mL of a saturated NaHCO_3_ solution. The organic phase was dried with MgSO_4_ and filtered. The solvent was removed using a rotary evaporator, and solvent residues were further removed in a vacuum oven at 50 °C. Activated molecular sieves (4 Å) were then added, and the dicarbonate was transferred to an argon-filled glovebox for storage until used. ^1^H NMR analysis was used to confirm the structure of the synthesized dicarbonate in addition to solvent removal. See the SI for the ^1^H NMR spectra.

Prior to mixing the electrolytes, the dicarbonates were filtered through PTFE syringe filters. LiTFSI was added at different molar ratios *r* = [LiTFSI]/[C=O] ratios (*r* = 1:20, 1:15, 1:10, 1:6, 1:3 = 0.05, 0.075, 0.1, 0.167, 0.33) referring to the molar concentration of LiTFSI and carbonate groups, respectively. Note that the dicarbonates have two carbonate groups per molecule compared to one in DMC/DEC. As an example, 0.52 g of LiTFSI was added to 2 g of PE to obtain *r* = 0.1. The electrolytes were left under magnetic stirring until fully dissolved (for several hours for the dicarbonates). For the dicarbonates with the highest salt concentrations (*r* = 0.167, 0.33), the mixture was heated to 50 °C to facilitate dissolution. For *r* = 0.1, the molar concentrations for EM, PM, EE, and PE were 1.1, 1.0, 0.9, and 0.9 M, respectively.

### Physical Characterization (DSC, TGA)

The thermal transitions were characterized using a DSC1 instrument from Mettler Toledo. Approximately 10 mg was added to an aluminum pan and experiments were performed under constant nitrogen flow (50 mL min^–1^). An initial heating step was employed at 10 °C min^–1^, up to 80 and 40 °C for DMC and DEC. Subsequently, a cooling step to −80 °C and a heating step to 80 °C, both at 5 °C min^–1^, were employed.

The thermal stability was characterized using a TGA1 instrument from Mettler Toledo. Approximately 15 mg was added to an open alumina pan and heated from 30 to 500 °C at a heating rate of 10 °C min^–1^ under nitrogen flow (50 mL min^–1^).

### Viscosity

The viscosity was determined by using a cone and plate viscometer CP1 (TQC Sheen) equipped with a 0–10 P cone set at a 750 rpm rotation speed. The temperature was varied by using the built-in programmable heating plate.

### Density

The density of the dicarbonates was determined by measuring the mass inside a 1 mL volumetric flask. Triplicates were taken for each dicarbonate.

### Electrochemical Impedance Spectroscopy (EIS)

A VMP3 Potentiostat from Biologic was used to perform EIS within the frequency range of 1 MHz–1 Hz with an AC amplitude of 10 mV. To measure the ionic conductivity, a Swagelok-like cell containing two blocking electrodes was used, as described elsewhere.^28^ A small amount of liquid electrolyte was added to one blocking electrode, and a 250 μm PTFE spacer was used. For the temperature sweeps, the cells were placed in a temperature chamber and allowed to equilibrate for at least 30 min at each temperature. Triplicates were taken at each temperature and doublets at each salt concentration.

### Fourier Transform Infrared Spectroscopy

Fourier transform infrared spectroscopy (FTIR) analysis was performed using a PerkinElmer Spectrum 100 instrument in attenuated total reflection (ATR) mode equipped with an MKII Golden Gate ATR accessory (Specac Ltd.). Sixteen scans were recorded with a resolution of 4 cm^–1^ for each spectrum. Electrolytes were prepared in an argon-filled glovebox and opened immediately before measurements were taken. A droplet of electrolyte was added onto the crystal, and a glass plate was placed on top.

### Molecular Dynamics Simulations

All-atom classical molecular dynamics (MD) simulations were performed to analyze the Li^+^ solvation structure and its dynamical properties. The atomic interactions within each molecular component and the intermolecular interactions were described by the nonpolarizable Generalized Amber Force Field (GAFF).^29^ The charges were calculated via the CHELPG method^30^ after DFT-based molecular optimization, followed by single-point calculations under the B97-3c/def2-TZVP level of theory and CPCM implicit solvation^31^ for DMC (dielectric constant, ε = 3.087^32^ and refractive index *n* = 1.368^33^). Those charges were employed as atomic point charges for the MD model, and for both cations and anions, they were scaled to 0.8 *e*, where *e* is the unit charge. This method has been extensively used in other works^34−37^ to mimic the polarization effects introduced in the systems with charged components; e.g., electrolytes. The number of molecules is shown in Table S2 in the SI. Figure S8 depicts the molecules (DMC, DEC, and dicarbonates) after DFT-based optimization, which were used as initial configurations for MD simulations. The systems were composed as shown in Table S2, and the Packmol software^38^ was employed to assemble them in a suitable simulation box. MD simulations were performed by Gromacs v. 2022.2,^39−42^ while DFT calculations were done in ORCA v. 5.0.3.^43,44^ The force field parameters were assembled through the AnteChamber PYthon Parser interfacE (ACPYPE) tool^45^ whose van der Waals and Coulomb interactions were scaled by 0.50 and 0.83, respectively, and their cutoff distance was set to 1.2 nm. The spatial distribution function (SDF) was analyzed by TRAVIS.^46^

MD simulations were carried out by the following protocol. First, energy minimization was done by two separate steps: steepest descent followed by conjugated gradient, where at every 40 steps, a steepest descent-based step was done. During the equilibration, a leapfrog stochastic dynamics (SD) simulation was performed for 2 ns at 700 K and 1 bar (time constant of 0.5 and 5.0 ps for velocity rescaling thermostat and Berendsen barostat,^47^ respectively). A Maxwell’s velocity distribution^48^ was assigned to the atoms at 700 K. From this point, an annealing simulation was performed from 700 K down to 298 K for 3 ns at 0.1 K/ps decreasing rate, which was followed by a canonical simulation for 1 ns at 298 K. Before the production phase, an NPT equilibration ran for 10 ns using a Parrinello–Rahman barostat^49^ at 1 bar and time constant of 5.0 ps. Production simulations were done for 400 ns at the same NPT ensemble as the previous step, and the energetics and the trajectory frames were saved every 250 steps. The time step was set to 2 fs.

## Results and Discussion

### System Description

**Scheme 1:**
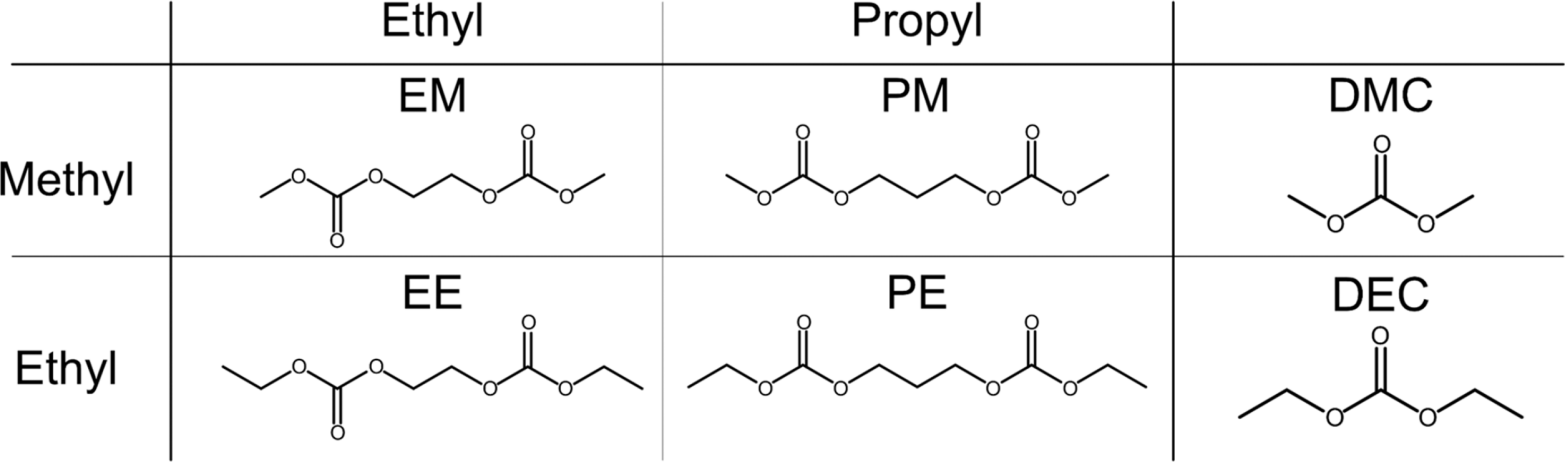
Chemical Structures of Dicarbonates and Linear Carbonates Investigated in This Study

The four dicarbonate solvents that were investigated in this study can be seen in Scheme 1. The structures create a matrix based on the two parameters being varied. First, the end group was either ethyl or methyl, to mimic the difference between DMC and DEC. Second, the spacer length between the two carbonate groups was varied, from two to three carbons. The dicarbonates are denoted by two letters: the first one denotes the end group length (E for ethyl or M for methyl) and the second one for the spacer length (E for ethyl or M for methyl). Two of these dicarbonates, EE and EM, correspond to two previously investigated dicarbonates, DEOHC and DMOHC,^23^ while PE and PM, to the best of our knowledge, are new for use in battery electrolytes.

A first step in the investigation involved developing a synthesis route for the four dicarbonates. A detailed description of the synthesis is included in the Experimental Section. The simple one-step reaction is based on the acylation of 1,3-propanediol or ethylene glycol with either ethyl or methyl chloroformate. The H^1^-NMR spectra of the four purified dicarbonates are shown in Figure S1, showing clean spectra without byproducts. Alternative synthesis routes utilizing DMC and DEC instead of chloroformates have been explored previously and could be a promising and sustainable way to synthesize these dicarbonates in the future.^50−52^ Electrolytes containing the dicarbonates were produced by dissolving LiTFSI at several salt concentrations, denoted *r* = [LiTFSI]/[C=O] = 1:20, 1:15, 1:10, 1:6, 1:3 = 0.05, 0.075, 0.1, 0.167, 0.33. LiTFSI was chosen over LiPF_6_ due to the higher thermal stability of the former. Using LiTFSI, however, could cause issues with the corrosion of the aluminum current collector which needs to be considered in a full cell configuration. This issue can, for example, be mitigated with lowering the upper cutoff voltage proposed by Taskovic et al.^3^

### Physical Properties

As a baseline for understanding the suitability of the four dicarbonates as electrolyte solvents, some physical properties were determined experimentally. These are summarized in Table 1. An important aspect in the development of novel electrolytes is at which temperature thermal transitions (melting point, etc.) occur. To maximize the operating window, a low melting point and a high boiling point are preferred. It is also well established that the addition of a salt (i.e., LiTFSI) suppresses crystallization, lowering the melting point of the electrolyte compared to the pure solvent. In addition, mixtures of multiple carbonate molecules (EC together with a linear carbonate) lead to a further reduction of the melting points compared to the pure carbonates.^53−55^ In Figure S3, the DSC thermographs of the pure dicarbonates as well as the electrolytes with *r* = 0.1 are presented. Three of the dicarbonates exhibit clear exothermic crystallization peaks and endothermic melting points. EM has the highest melting point (18 °C), rather close to 16 °C which was previously reported by Taskovic et al.^23^ Furthermore, EM displays two crystallization peaks, indicating the presence of two different crystalline structures. Notably, EE does not exhibit any thermal transitions down to −80 °C, also in line with Taskovic et al.^23^ Upon the addition of LiTFSI, the crystallization is fully suppressed in PE. EM and PM still crystallize, but the melting point is pushed to a lower temperature. It is worth noting that Zhang et al. did not observe a melting point for EM (DMOHC) with 1 M LiPF_6_ which may be due to the use of a different salt, or the higher scan rate (10 °C min^–1^ compared to 5 °C min^–1^ used in this study). Generally, the dicarbonates with ethyl end groups have lower/no melting point compared to the dicarbonates with methyl end groups, which is likely due to the methyl-containing dicarbonates being able to pack into crystal structures more easily considering their smaller size. This is also reflected in the density measurements (see Table 1) where EM and PM have higher densities than EE and PE. These results also correlate well with DEC and DMC, as DEC has a lower melting point (−43 °C) and density than DMC (−3 °C).^53^

**Table 1:** Physical Properties of Linear Carbonate Solvents and Electrolytes Using LiTFSI

				with LiTFSI (*r* = 0.1)	
	*M* (g mol^–1^)	density (g mL^–1^)	melting point (°C)	melting point (°C)	TGA onset point (°C)
PE	220.2	1.13	1.4		184
PM	192.2	1.22	3.6	0.2	170
EE	206.2	1.15			180
EM	178.2	1.26	18	12.6	175
DEC	118.1^a^	0.98^a^	–43^a^		
DMC	90.1^a^	1.07^a^	3^a^	–3	

a From Smart et al.^53^

**Figure 1:**
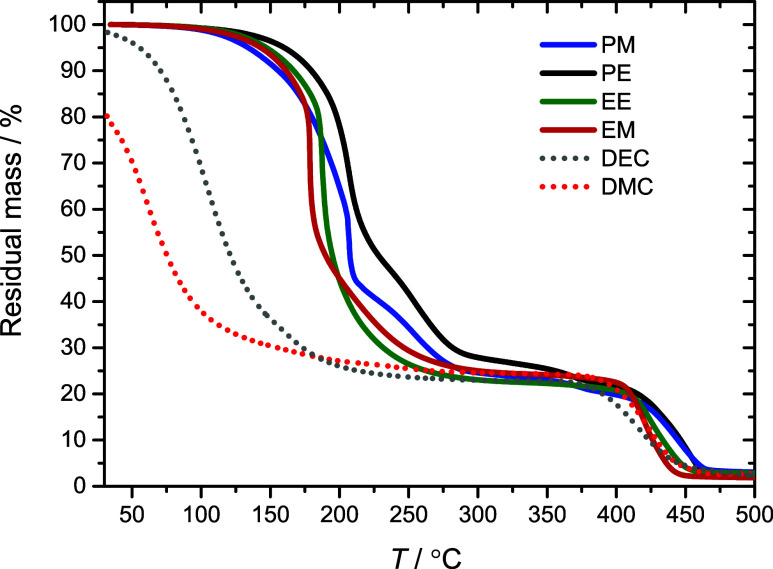
TGA thermogram with a heating rate of 10 °C min^–1^ for linear carbonate and dicarbonate electrolytes.

The thermal stability of the electrolytes is also an important characteristic. Figure 1 shows TGA curves for four dicarbonate electrolytes, with DMC and DEC as references. Notably, about 20% of DMC evaporates prior to the first measurement point due to the high volatility of the solvent at ambient temperature. The curves show a clear enhancement of thermal stability when moving from the single carbonate solvent to the dicarbonate structures, in line with previous reports.^24^ Moreover, the dicarbonates start to evaporate and break down at similar temperatures, with PM and EM displaying the lowest onset point. The high thermal stability of LiTFSI is also clear, degrading above 400 °C (see Figure S4). A temperature step test was also performed on PE to counteract kinetic effects that may underestimate the volatility of solvents (Figure S5). The PE electrolyte was first held at 100 °C for 1 h, showing minimal weight loss (about 4 wt %). At 150 °C, a more significant weight loss was observed after 1 h (about 35 wt %). It is worth noting that the thermal stability of the linear carbonates can be further improved by increasing the chain length to oligomeric and polymeric species.^28,56^ However, the largest improvement in thermal stability is made when shifting from single carbonate solvents to dicarbonates.^28^ Considering the high cycling stability of a dicarbonate electrolyte at 85 °C,^23^ one may consider this sufficiently stable for many battery applications.

### Ion Transport

**Figure 2:**
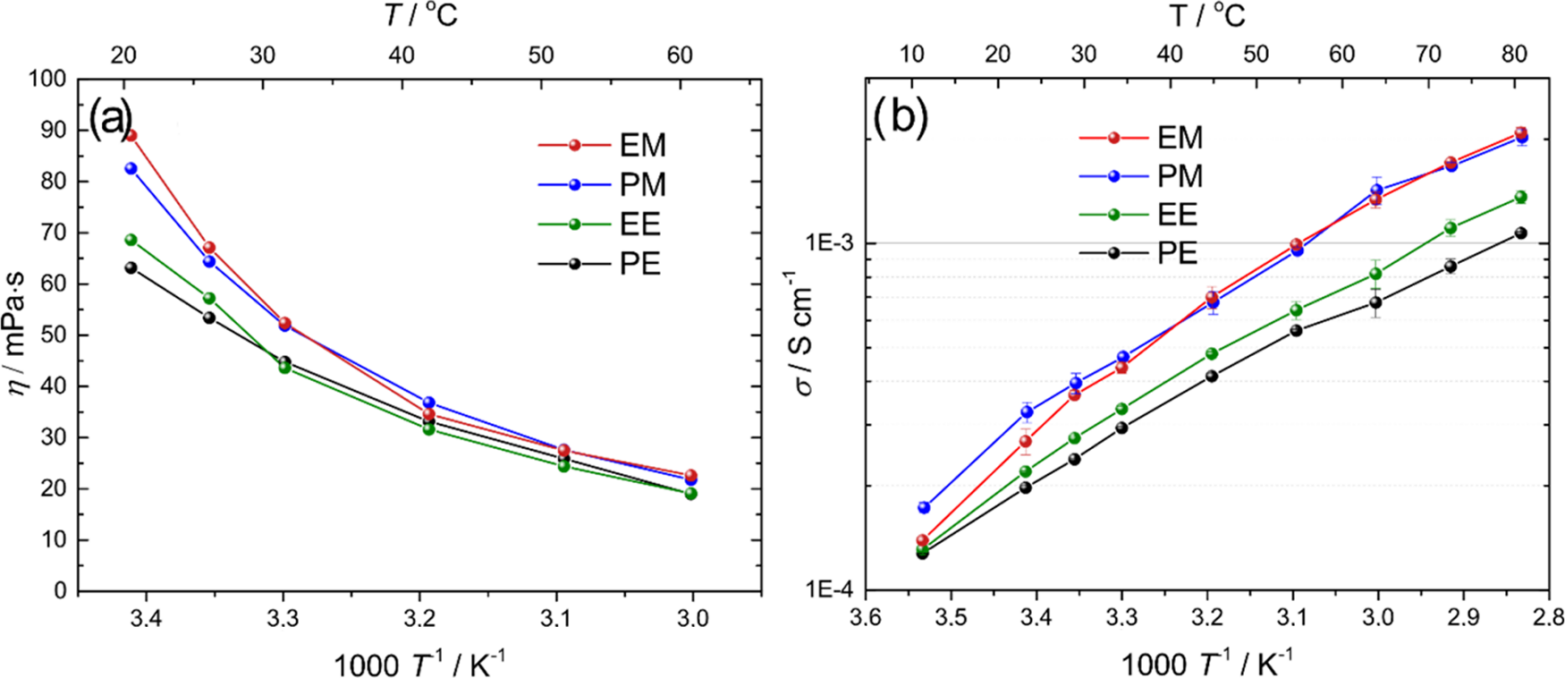
(a) Viscosity and (b) total ionic conductivity as a function of temperature for four dicarbonate electrolytes. A constant salt concentration was set at *r* = 0.1. Lines are added to guide the eye.

The viscosity at 20 °C of all dicarbonate electrolytes containing LiTFSI (*r* = 0.1) is significantly higher than most typical liquid electrolytes, in line with previous reports.^23,24^ The viscosity decreases rapidly upon increasing temperature, showing a Vogel–Fulcher–Tammann (VFT)-like dependence (see Figure 2a). Interestingly, the viscosity at ambient temperature of the electrolytes follows the order EM > PM > EE > PE, which is the inverse order of molecular size. This suggests that the smaller dicarbonates form stronger intermolecular interactions, which leads to a higher viscosity. This is also well aligned with the DSC and density measurements. In addition, the end group seems to have a larger effect than the spacer. It should be noted that the differences between the dicarbonates are rather small compared to the difference between DMC and DEC, which have significantly lower viscosities.^23^

**Figure 3:**
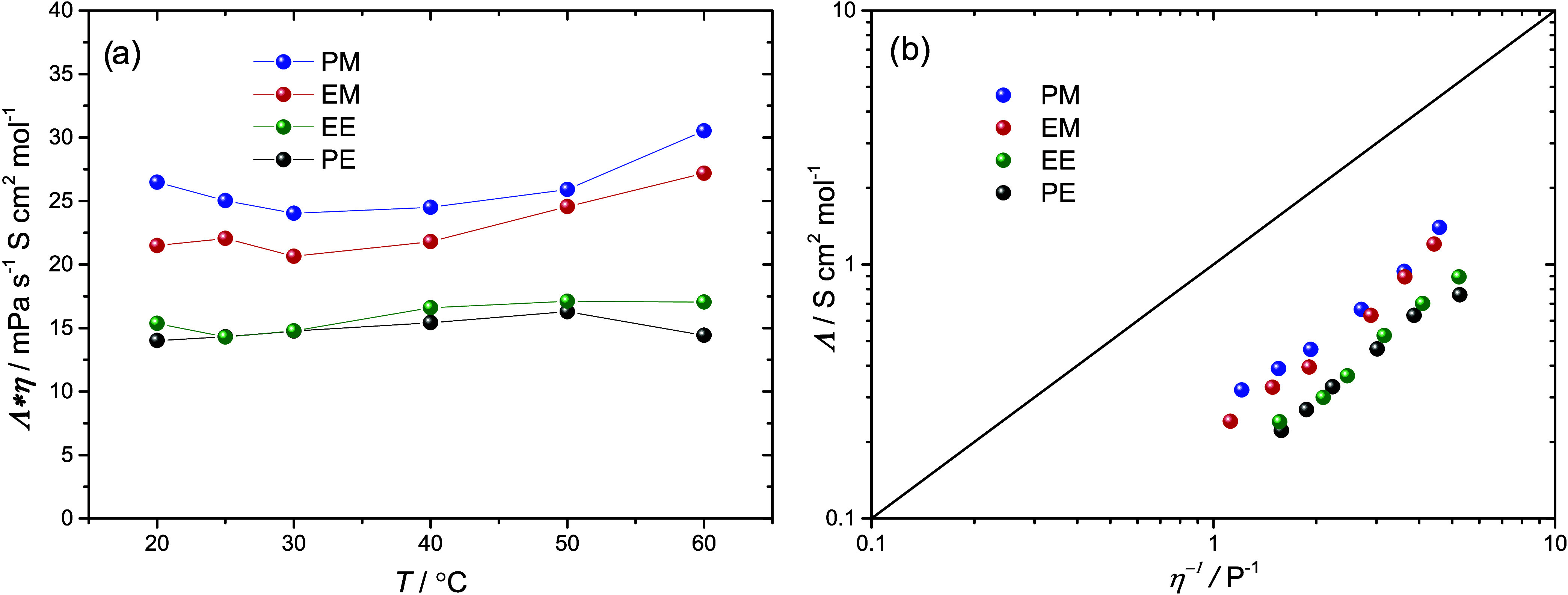
(a) Product of the molar conductivity and viscosity as a function of temperature and (b) Walden plot. A constant salt concentration was set at *r* = 0.1. The solid line in (b) represents the “ideal” KCl line.^61^

The ionic conductivities (σ) of the four dicarbonate electrolytes were measured for a range of temperatures; see Figure 2b. The conductivity is around 1 order of magnitude lower than pure DEC and DMC electrolytes,^57^ but similar to previously reported values for EM-based electrolytes.^23,24^ Once again, the dicarbonates fall into two groups, with PM and EM generally displaying a similar σ, higher than those for EE and PE. The higher σ values of PM and EM correlate well with DMC displaying higher σ than DEC.^57^ A useful way to compare liquid electrolytes is to analyze the product of molar conductivity and viscosity (Λ·η), as seen in Figure 3a. The product indicates the ionicity of the system, with a higher value reflecting a higher ionicity.^58,59^ A higher ionicity is usually attained in more polar solvents, which can dissociate the salt better. In this case, PM has a slightly higher ionicity than EM, which, in turn, is clearly higher than EE and PE. Although the polarity of the dicarbonates may differ slightly, the difference is rather small. In addition, coordination effects can undoubtedly play a role, where the methyl end groups may allow for improved ion solvation (larger salt dissociation) compared with the ethyl end groups. This has previously been observed when comparing DMC and DEC, contributing to the higher ionic conductivity observed in DMC compared to DEC.^57,60^ A Walden plot is also shown in Figure 3b. This shows that all electrolytes are below the “ideal line” for fully dissociated salts with completely coupled viscosity and ionic conductivity. This is not uncommon for nonaqueous liquid electrolytes, but suggests the presence of ion pairs and nonideal ion transport.^58,59^

**Figure 4:**
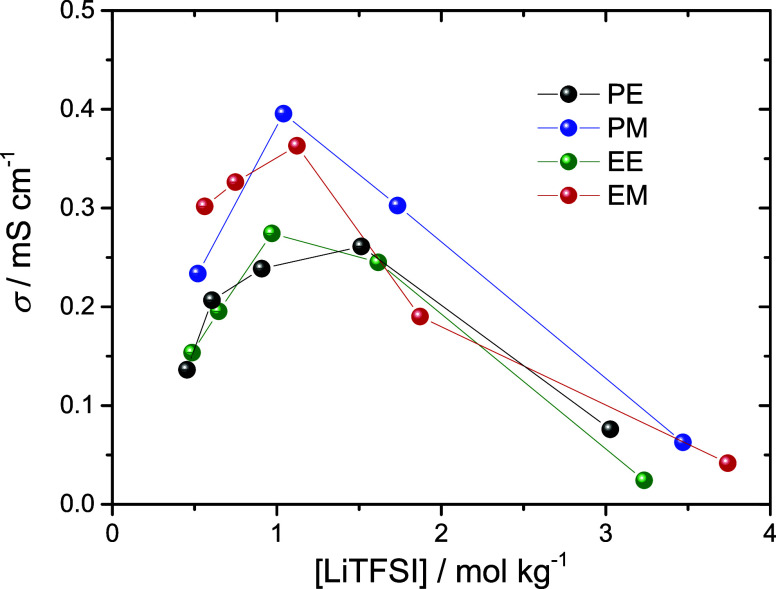
Total ionic conductivity at 25 °C as a function of the LiTFSI content. The concentrations were set to constant *r* = [LiTFSI]/[C=O] = 0.05, 0.075, 0.1, 0.167, 0.33.

In Figure 4, the ionic conductivities of the four dicarbonates as a function of LiTFSI concentration are presented. For liquid electrolytes, σ typically increases initially upon increasing salt concentration due to the increasing presence of charge carrier. Eventually, the viscosity starts to dominate upon which σ decreases with the addition of salt. In EC-containing electrolytes (i.e., EC/DMC), the highest conductivity is typically reached at moderate concentrations (1 M). This is partly due to the high dielectric constant of EC, which means that the permittivity decreases with the addition of salt. In pure linear carbonates (i.e., DMC or DEC) which have a low dielectric constant, the permittivity increases with the addition of salt, and the maximum is reached at higher concentrations (2 M).^59^ For all dicarbonates, σ increases with an increasing LiTFSI content up to *r* = 0.1 (∼1 mol kg^–1^). The increase is however rather modest (maximum a factor of 2). When further increasing the LiTFSI content to *r* = 0.167 (1.5–2 mol kg^–1^), σ decreases for all dicarbonates except PE. When increasing salt content into the HCE regime (*r* = 0.33), the conductivity decreases dramatically for all dicarbonate systems. Both EE and EM seem to reach their solubility limit, with the electrolyte solidifying at ambient temperature. This may be due to the short spacer in EE and EM, which possibly constrains ion dissociation. In general, the maximum conductivity seems to be reached at a lower concentration than DMC or DEC. These results motivate further investigation of the coordination structure of the dicarbonates.

### Ion Coordination

To evaluate the coordination environment of LiTFSI in the different dicarbonates, ATR-FTIR spectra were acquired for all dicarbonates, as well as DMC and DEC at various concentrations of LiTFSI. A peak for uncoordinated C=O can be seen at around 1750 cm^–1^, and the shifted peak at around 1720 cm^–1^ represents Li^+^ coordinated C=O (see Figure S6). By deconvolution of the two C=O bands, the relative quantities of coordinated and uncoordinated carbonates can be estimated.^62−65^ From this, the coordination number (CN) can be found by multiplying this by the [Li^+^]/[C=O] ratio. This has previously been used to determine CN in polymeric and oligomeric carbonates^64,66^ and the solvation number for carbonate solvents,^62,67^ which is the same as the CN for solvents with one carbonate per molecule.

**Figure 5:**
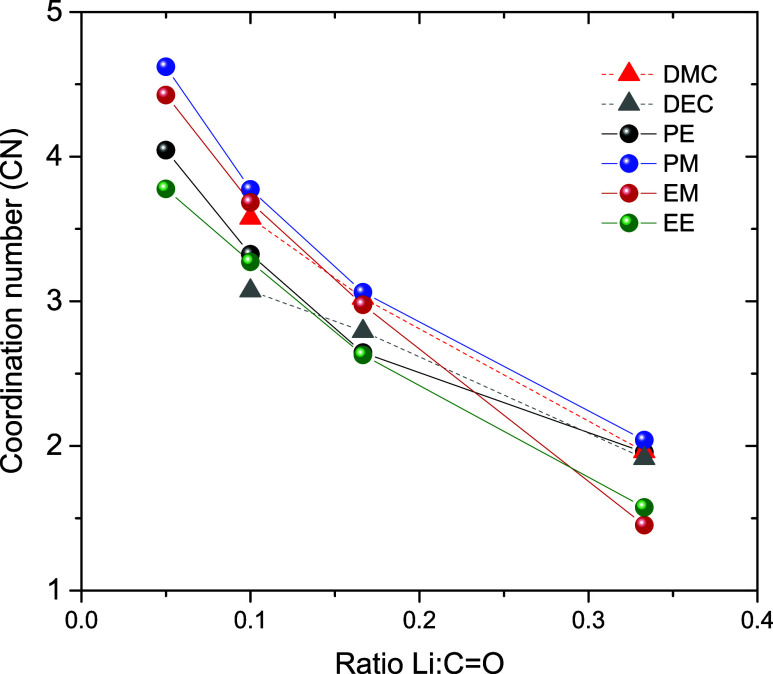
Carbonyl coordination number (CN) as a function of the salt:carbonyl ratio as determined by FTIR.

As previously established, however, the extinction factors of coordinating carbonyls and free carbonyls are different, leading to an overestimation of the CN if this is not considered.^62,67^ In Figure S7, the CN without using the extinction coefficient is shown, giving CN above 5 at a low salt concentration. To counteract this, the extinction factor ratios between coordinating and free carbonyls for DMC and DEC determined by Lim et al. were used.^67^ For the dicarbonates, the extinction factor ratio for DMC was used for EM and PM while the extinction factor ratio for DEC was used for EE and PE as they have the same end group. The corrected CN are displayed in Figure 5. With this correction, the CN for DMC and DEC better match the values obtained in previous studies.^62,67^ The CN obtained for the dicarbonates is similar to those of DMC and DEC. EM and PM display a slightly higher CN than PE and EE, particularly at moderate salt concentration. This could explain the difference in ionicity from Figure 3. In general, the CN values of the different dicarbonates are within the same range (a maximum difference of 0.5 at *r* = 0.1) and similar to DMC and DEC. The exact CN is also dependent on the correct extinction coefficient, which in this case is only estimated from the linear carbonates. It is also worth noting the CN does not say much about the presence or absence of the anion. The shift of the carbonyl peak from carbonyls coordinating to solvent-separated Li^+^, and those which are coordinating to contact ion pairs (CIP) generally overlap.^67,68^

**Figure 6:**
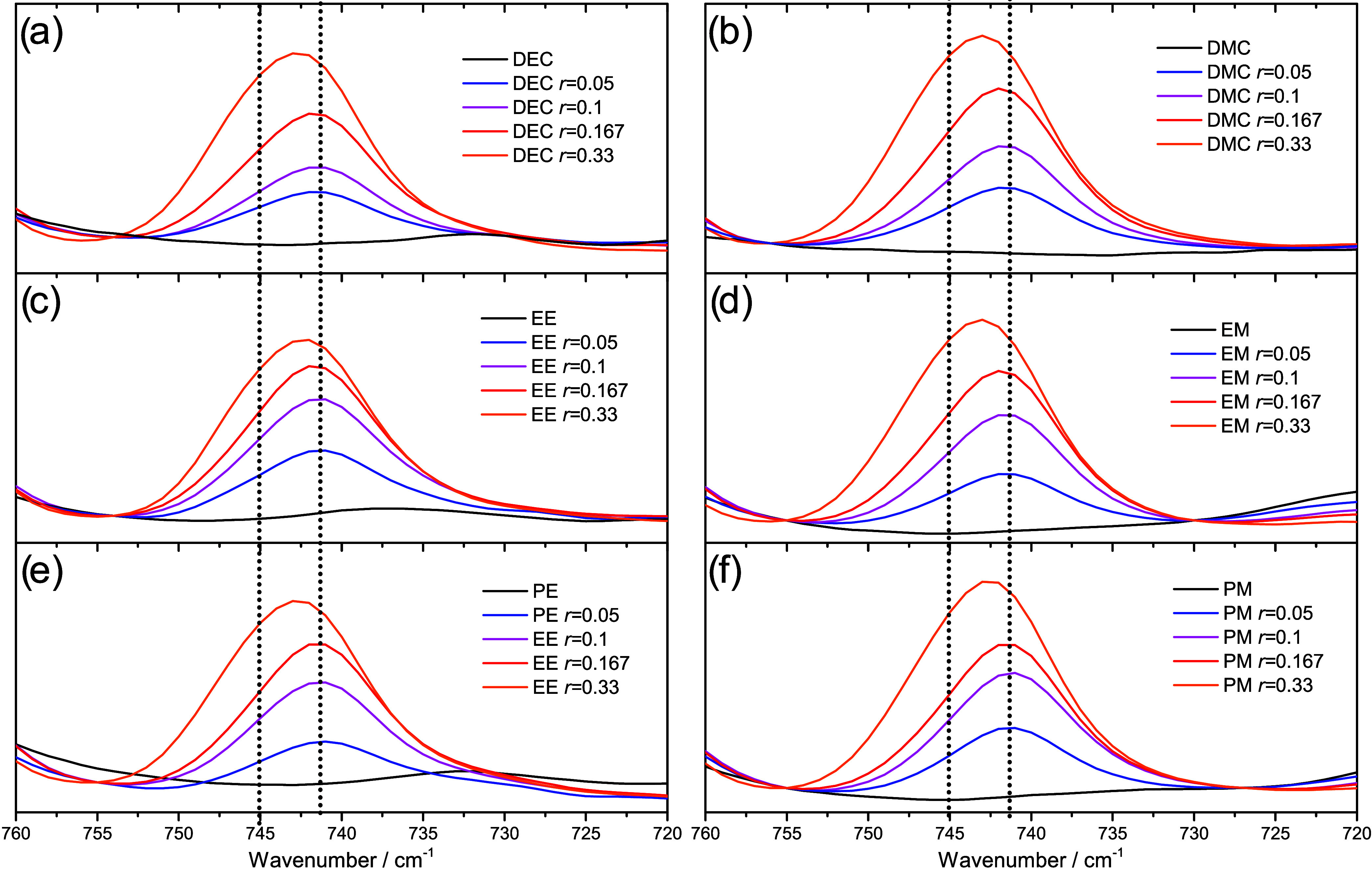
FTIR spectra for (a) DEC, (b) DMC, (c) EE, (d) EM, (e) PE, and (f) PM for the region 720–760 cm^–1^.

Apart from the carbonyl group, FTIR was also used to investigate the band corresponding to the TFSI anion. A band at around 740 cm^–1^ corresponds to “free” TFSI anions, which are dissociated. A shift to a higher wavenumber (745 cm^–1^) indicates a higher content of ion pairing and aggregates. Figure 6 shows the TFSI^–^ band for all carbonate solvents. Although a shoulder toward higher wavenumbers emerges when increasing salt concentration, the clearest shift in the peak is seen when moving to *r* = 0.33 for all carbonates. In addition, no clear differences could be identified when comparing the different solvents. A reason for this could be the presence of small overlapping bands for EE, PE, and DEC without the presence of LiTFSI.

### Computational Analysis

To further build an understanding of the coordination structure and ion transport in the dicarbonate electrolytes, molecular dynamics simulations were performed. Radial distribution functions (RDF) between Li^+^ and both carbonate atoms of dicarbonates, DMC and DEC, and the oxygen atoms of TFSI^–^ are shown in Figure S9. All of the analyzed systems show the main RDF peak at 2.1 Å, but the Li^+^–TFSI[O] pair also presents a second peak around 4.5 Å for all salt concentrations analyzed. These results indicate that TFSI^–^ exists in the same solvation shell composed of carbonates but may also form a second solvation shell 2.3 Å away from the first shell. The spatial distribution function (SDF) depicts colored surfaces (isosurfaces) showing the average position of the system’s components. In Figure S10, the SDFs show how the anions make part of the first solvation shell along with the dicarbonates regardless of the salt concentration.

**Figure 7:**
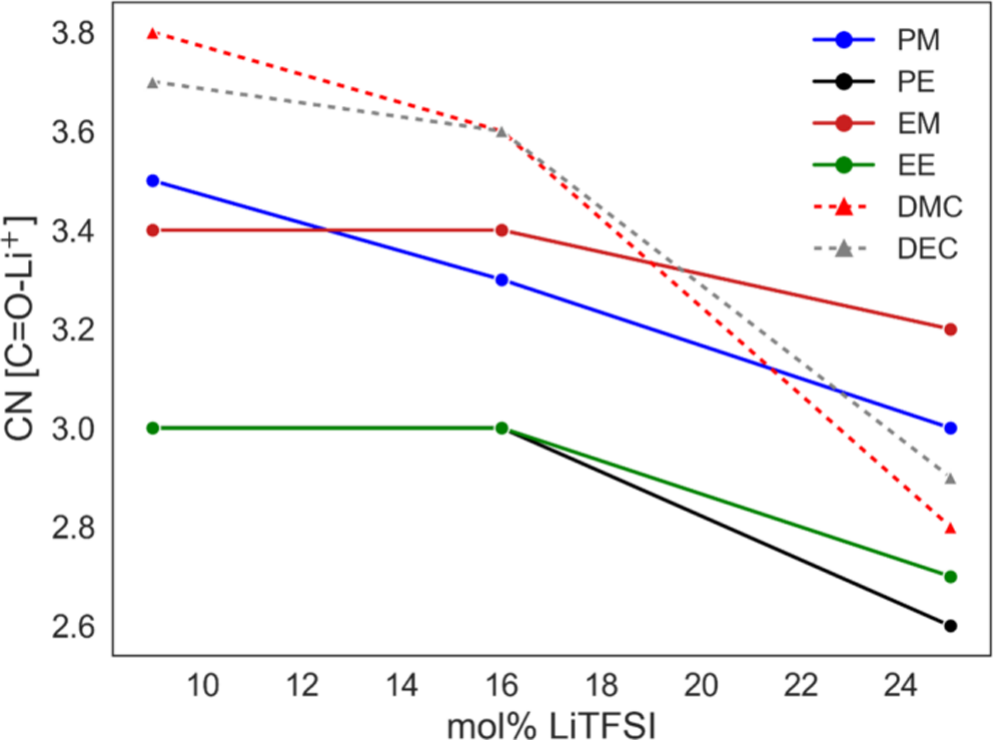
Coordination numbers (CN) of oxygen carbonate, with respect to the Li cation. These quantities were calculated at around the first RDF minima, about 3.0 Å, as can be seen in Figure S9. Note that 9, 16, and 25 mol % represent *r* = 0.05, 0.1, and 0.166 for dicarbonates and *r* = 0.1, 0.2, and 0.33 for DMC and DEC.

Based on the RDFs shown in Figure S9, the CN between the carbonate oxygen and Li^+^ for different salt concentrations was evaluated, as can be seen in Figure 7. Our simulated results for DMC are corroborated with results found in the literature for both LiTFSI^69^ and LiPF_6_.^57^ For DMC-LiPF_6_, there are also DFT-based results showing similar CN as found here for the systems with DMC.^70^ In DMC-LiPF_6_ systems, the coordination number is assumed to be four, comprising one anion and three linear carbonate,^57^ which was observed in our simulations but for TFSI, as can be seen in Figure S9. For DMC, as the salt concentration increases, the carbonyl CN decreases (from 3.8 to 2.8), which is also in reasonable agreement with experimental results^62,67^ and the one performed in this work. However, it is also important to consider artifacts that may appear due to the charges used in the atomic model as pointed by different studies.^36,71^

The dicarbonate systems differ from those of DMC and DEC in two clear ways. First, at low to moderate salt concentration, the Li^+^-carbonyl CN for all dicarbonates is slightly lower. This differs from the experimental results where the CN was similar or slightly higher, but the differences are small, as also observed experimentally. Second, the salt concentration seems to have a rather small impact on the CN, similar to what was observed with the ionic conductivity. The dicarbonates can also be grouped into their respective end groups, with PM and EM displaying higher Li^+^-carbonyl CN than PE and EE, as could be seen in the FTIR data.

When considering the CN for Li^+^–TFSI[O], the opposite trend is observed, as seen in Figure S11, EE and PE have the highest CN, while all dicarbonates display higher CN than DMC and DEC at low to moderate salt concentrations. At 25 mol %, the CN for DMC and DEC increases significantly to above 2 which is often observed when approaching the HCE regime. The clear presence of TFSI^–^ in the solvation shell even at a low salt concentration underlines the weak solvation power of these dicarbonates. This was also observed by Zhang et al. for EM when compared to EC/DMC, suggesting that it contributes to the formation of a strong SEI layer on Li metal.^24^ The presence of the TFSI^–^ will, however, also affect the lithium ion transport, which was therefore investigated further.

**Figure 8:**
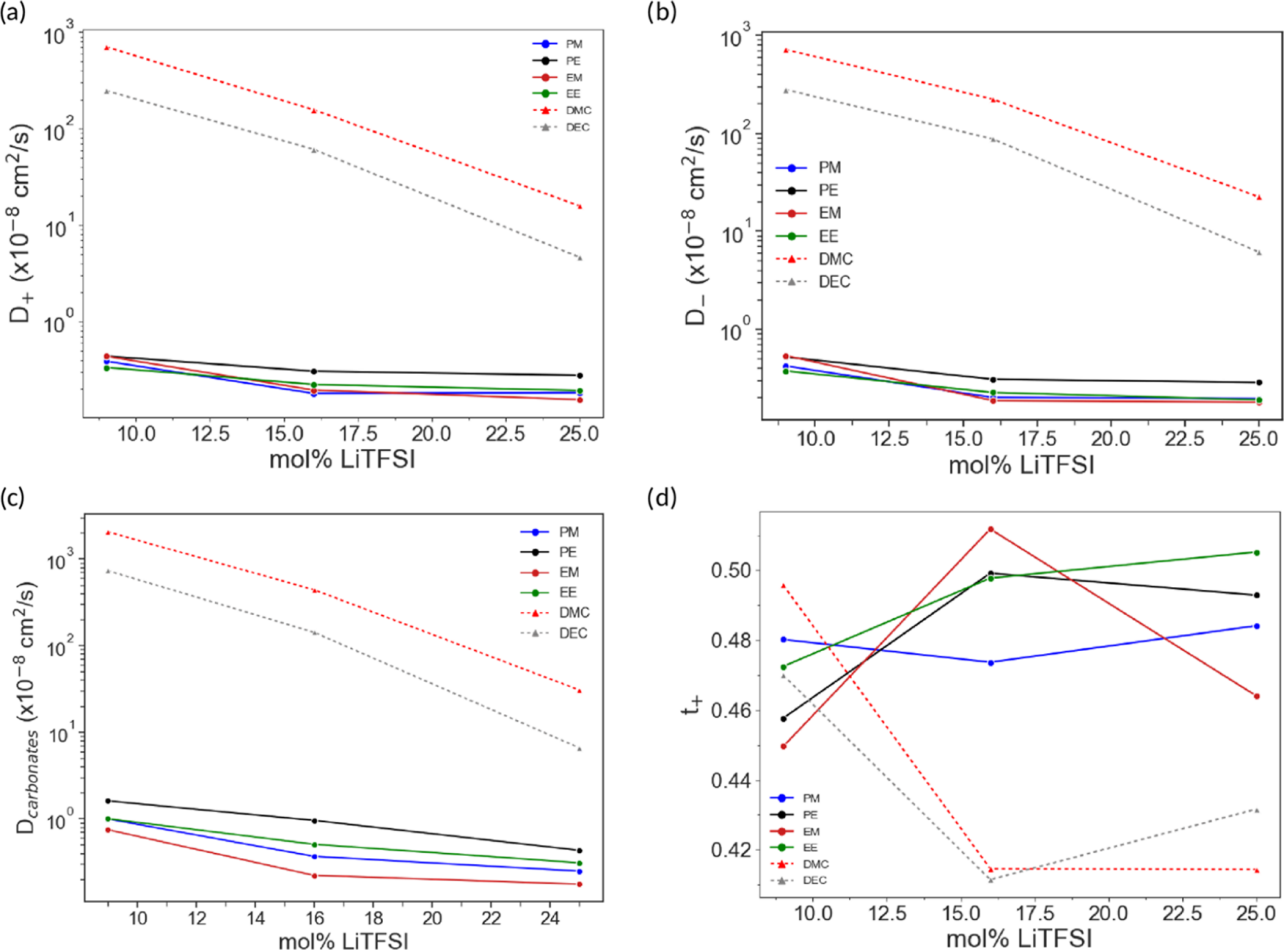
Diffusion coefficients (a) *D*_+_, (b) *D*_–_, and (c) *D*_carbonates_ for the different carbonate electrolytes (inset shows the dicarbonate electrolytes) and (d) transference number (*t*_+_) at different salt concentrations. Both *D*_carbonates_ and *D*_–_ are taken with respect to the molecule center of mass; the diffusion coefficient is calculated from the mean square displacement curves using the Einstein relation.

The diffusion coefficients for each component of the electrolytes and the transference number for Li^+^, calculated as *t*_+_ = *D*_+_/(*D*_+_ + *D*_–_), were also determined. Here, *D*_±_ is the diffusion coefficient for either Li^+^ or for TFSI^–^. Both *D*_carbonates_ and *D*_–_ are taken with respect to the molecular center of mass; the diffusion coefficient is calculated from the mean square displacement curves using the Einstein relation,^72^ where D is taken as the angular coefficient in the linear regime of the MSD curves. In Figure 8a–c, it is clear that the DMC-based systems display higher diffusion values than DEC, in line with previous studies.^54,57^ The DMC- and DEC-based systems present significantly higher values of *D*_+_, *D*_–_, and *D*_carbonates_ than the respective dicarbonates’ values, which is qualitatively in line with the high viscosity and low ionic conductivity of the dicarbonates experimentally found. However, *D*_±_ are more than 3 orders of magnitude higher in DMC than in the dicarbonates, which is not reflected in the difference in ionic conductivity. It is worth noting that *D*_±_ only represents the uncorrelated self-diffusion in the electrolytes, disregarding the correlated ion motion that may arise from contact ion pairs (CIPs) or clusters.^73^ This suggests that the MD simulations performed are only able to provide a trend that would indicate how the components move.

The ideal transference number, *t*_+_, for the dicarbonates (Figure 8d) was found to be as high as 0.5, which is higher than what is typical for liquid electrolytes and may be due to the formation of contact ion pairs (CIP).^74^ In general, the *t*_+_ of DMC and DEC are slightly lower, with the exception at 9 mol % salt concentration where they are in the same range. This, however, must be experimentally confirmed. Moreover, the simulated *D*_carbonates_ show overall higher values than the ion counterparts (Figure 8c), further indicating a nonideal ion transport and that either the salt was not dissolved by the solvents or the carbonates are moving together with CIP (forming aggregates).

**Figure 9:**
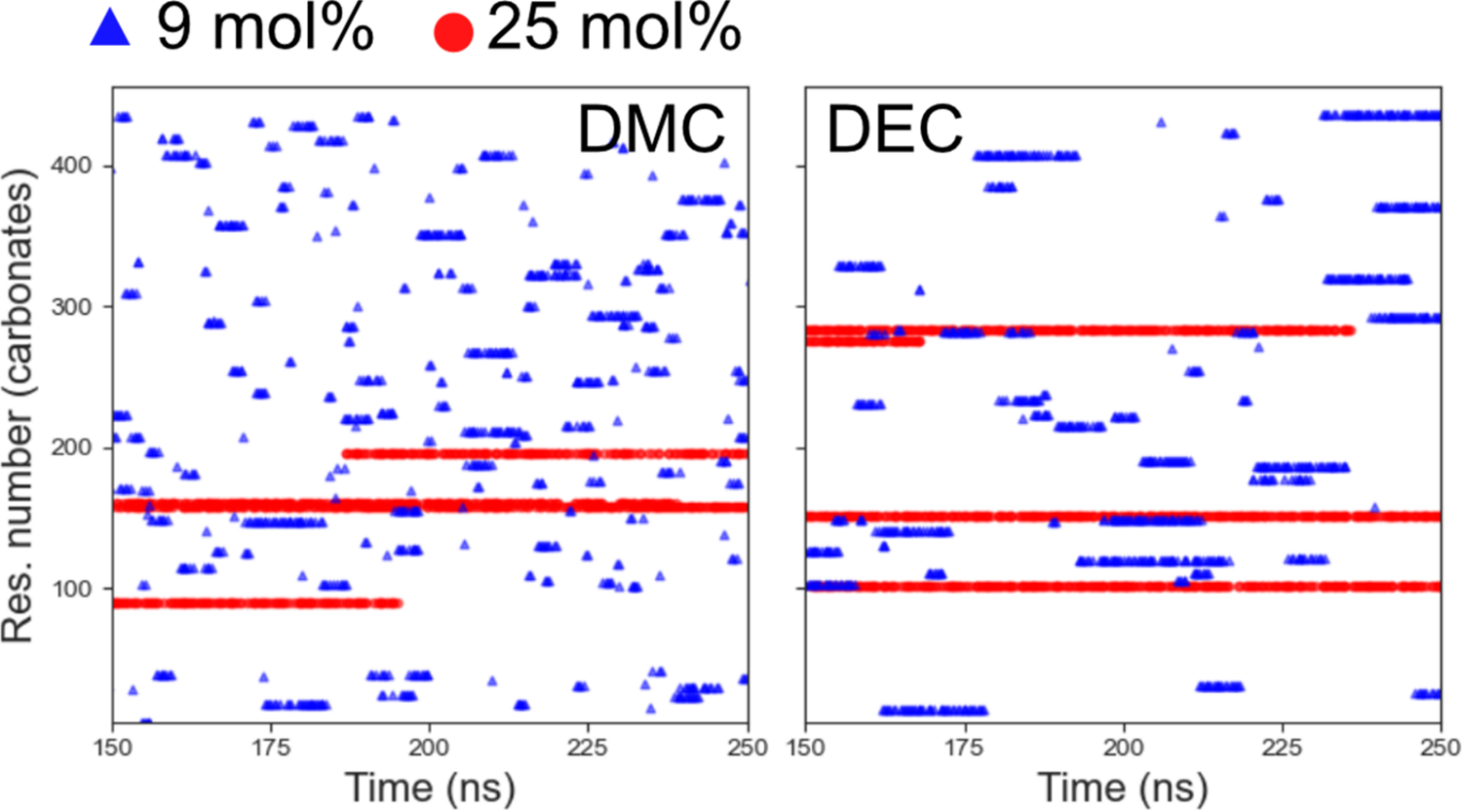
Time evolution plots for one Li^+^ in “contact” with DMC and one Li^+^ in “contact” with DEC, which means it is at a certain distance from the oxygen carbonyl (taken as the RDF maxima). If the distance between Li^+^ and oxygen carbonyl is less than 2.1 Å, a dot is plotted. The Li^+^ contacts to the carbonates were taken every 250 ps. Blue triangles and red triangles stand for salt concentrations of 9 and 25 mol %. The residue numbers on the vertical sites are numbers assigned to each carbonate molecule (375 and 455 molecules for 9 and 25 mol %, respectively). The plot for the whole simulation time (400 ns) can be seen in Figure S12.

**Figure 10:**
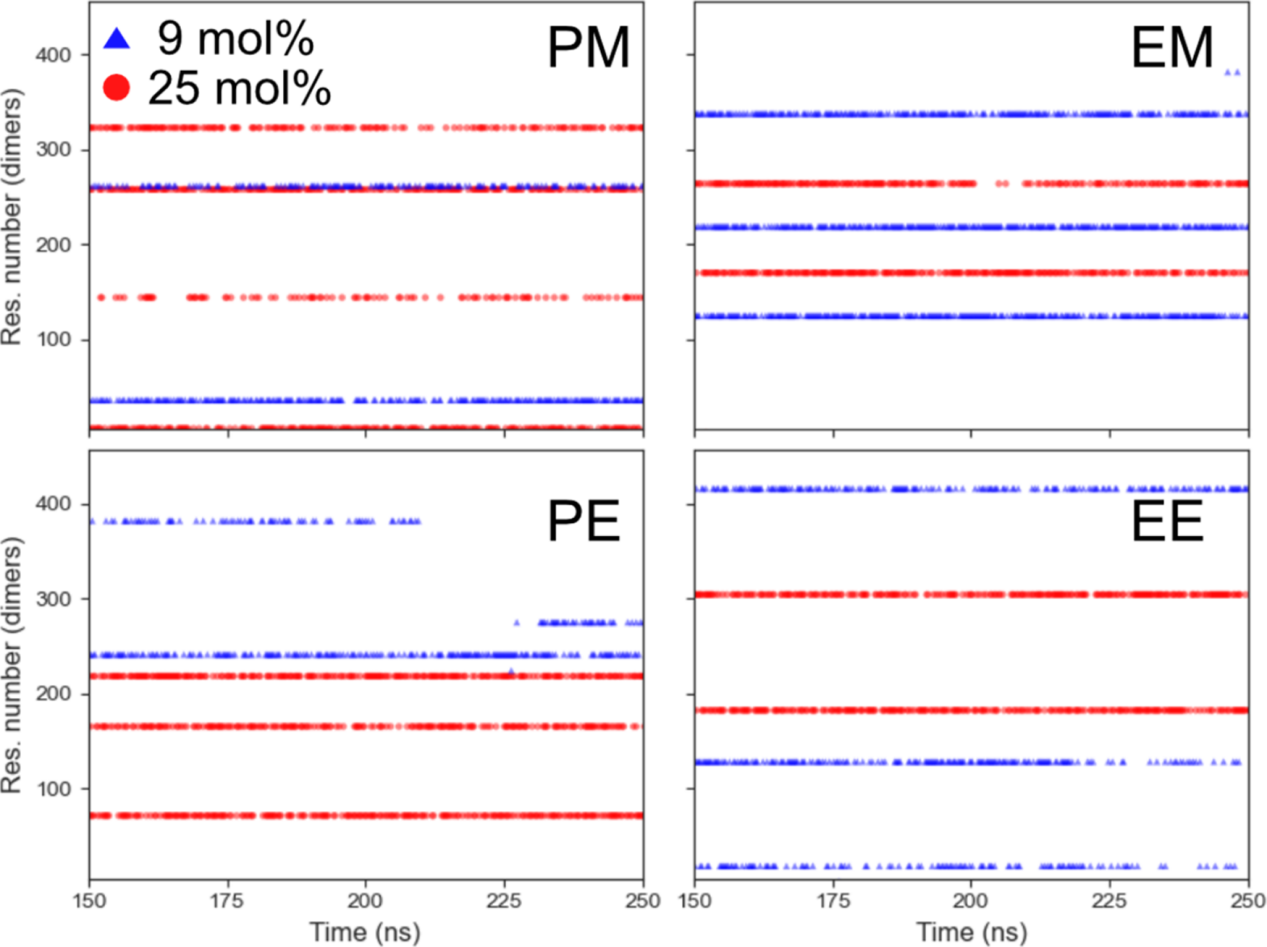
Time evolution plots for a Li^+^ in “contact” with the dicarbonates. The residue number on the vertical sites are numbers assigned to each carbonate molecule (375 and 455 molecules for 9 and 25 mol %, respectively). The plot for the whole simulation time (400 ns) can be seen in Figure S13.

In order to better understand the transport mechanism of Li^+^ in the carbonate electrolytes, the changes in the ionic coordination environment were analyzed in the time evolution plots shown in Figures 9 and 10. In liquid electrolytes, Li^+^ transport can occur via rapid solvation exchange (independent movement), which in a time evolution plot would be represented by multiple points throughout the plot. Alternatively, the ion is transported via co-diffusion with solvent molecules (vehicular transport) or counterion (CIP), which would instead be represented by continuous lines within a certain region in the time evolution plot.^70^

Figure 9 shows that for DMC at the lower salt concentration, the Li^+^ generally spends a short time in a certain region, indicating rapid solvation exchange. For DEC at the same salt concentration, Li^+^ is bound to the same DEC molecules for a slightly longer time. This could be due to the bulkier DEC being more sterically hindered compared to DMC. In general, previous studies have shown that DEC and DMC have a highly dynamic solvation shell, more so than cyclic carbonate.^68^ For a higher salt concentration, Li^+^ stays longer within a certain region, bound to specific carbonate molecules. This can be explained by the restricted mobility of the molecules, which are to a much greater extent already bound to Li^+^. This leads to more correlated ion transport.

For the dicarbonates, similar behaviors are seen for both salt concentrations (Figure 10). Most of the time, Li^+^ binds to up to three dicarbonate molecules (inferred from the CNs shown in Figure 7), going back and forth between these same molecules, thereby mainly staying within the same region of the plots. From visual analysis of the simulation time, some additional observations were made with regard to the ion transport mechanism (see Figures S12 and S13). The PE and PM seem to mainly form coordination structures with two carbonyls and one anion, while the EM and EE systems often involve two anions. The visualizations also showed that the longest dicarbonates (e.g., PE) wrap around the Li^+^ to a greater extent, while the shortest dicarbonates (EM) do not. For all dicarbonates, it is rare that both carbonyls coordinate with the same Li^+^. This means that there often are “dangling” carbonyl groups that are facing out from Li^+^, hindering new solvents from entering the solvation shell. To summarize, both the linear carbonates DMC and DEC as well as the dicarbonates display similar and rather low carbonyl CN (compared to cyclic carbonates^62,67^). However, the ion transport mechanism differs between the linear carbonates and the dicarbonates. While DMC and DEC display rapid solvent exchanges at a low salt concentration, the dicarbonates do not.

## Conclusions

To conclude, the ion transport in dicarbonates with varying end groups and spacer lengths was investigated and compared to the linear carbonates DMC and DEC. All dicarbonates, regardless of the structure, show promising thermal stabilities, but suffer from high viscosity and low ionic conductivity at room temperature. The ionic conductivity of the dicarbonates with methyl end groups was higher than those with ethyl end groups. This was attributed to a more preferential ion coordination in these dicarbonates, which was supported by MD simulations. RDFs reveal that the carbonyl CN is slightly lower than those of DMC and DEC. More significantly, the RDFs for TFSI^–^ display two clear peaks, with Li^+^–TFSI[O] CN as high as 2 for dicarbonates, indicating a significant presence of CIP.

The ion transport mechanism was followed in time evolution plots, which reveal that rapid solvent exchange in the coordination shell around Li^+^ occurs in DMC and DEC at a low salt concentration. When the salt concentration increases, the ion solvent exchange decreases significantly, and Li^+^ moves in a more correlated motion. For the dicarbonate electrolytes, the exchange of solvents around Li^+^ is limited, and Li^+^ mainly moves together with coordinating dicarbonate molecules and TFSI^–^.

These results underline that the solvent structure can be tuned to achieve a weaker solvation power, allowing for the participation of the anion in the solvation shell. Specifically, electrolytes using dicarbonates display many of the same traits as HCE (high population of CIP, high thermal stability, etc.) but at moderate salt concentration. This is also coupled with a nonideal ion transport, ultimately limiting their use to low-rate applications at room temperature. Future studies should explore which effects dicarbonates have on the solvation structure and ion transport in mixed electrolytes. The possibility of new dimers with other functional groups could also be an interesting pathway for future research.

## Supporting Information Available

Synthesis protocol; NMR spectra; DSC thermograms; TGA thermograms; FTIR spectra; coordination number calculation; and MD simulations (PDF)

## Abbreviations

DMC: dimethyl carbonate
DEC: diethyl carbonate
EMC: ethyl methyl carbonate
EC: ethylene carbonate
DMOHC: dimethyl-2,5-dioxahexane carboxylate
SEI: solid-electrolyte interphase
HCE: highly concentrated electrolytes
LHCE: iocalized highly concentrated electrolytes
LE: liquid electrolyte
LiTFSI: lithium bis(trifluoromethanesulfonyl)imide
LiFSI: bis(fluorosulfonyl)imide
LiPF_6_: lithium hexafluorophosphate
LMB: lithium metal batteries
CEI: cathode-electrolyte interphase
MD: molecular dynamics
DFT: density functional theory
CHELPG: CHarges from ELectrostatic Potentials using a Grid
DSC: differential scanning calorimetry
TGA: thermal gravimetric analysis
EIS: electrochemical impedance spectroscopy
FTIR: Fourier transform infrared spectroscopy
η: viscosity
σ: ionic conductivity
CN: coordination number
CIP: contact ion pairs
RDF: radial distribution functions
SDF: spatial distribution function
