# Supplementary material for: Understanding Ion Transport in Alkyl Dicarbonates: An Experimental and Computational Study
Source: ACS Phys Chem Au. 2024 Nov 10;5(1):80–91. doi: 10.1021/acsphyschemau.4c00078 (PMC11758495; doi:10.1021/acsphyschemau.4c00078)
Supplement: Supplementary file 1 — pg4c00078_si_001.pdf [file pg4c00078_si_001.pdf]

Understanding ion transport in alkyl dicarbonates: an experimental and computational study

Samuel Emilsson[a], *Marcelo Albuquerque*[b,c], *Pernilla Öberg*[a], *Daniel Brandell*[c], *Mats Johansson*[a*]

[a]Department of Fibre & Polymer Technology, Division of Coating Technology, KTH Royal Institute of Technology, SE-100 44 Stockholm, Sweden

[b]Institute of Physics, Universidade Federal Fluminense (UFF), Praia Vermelha Campus, Boa Viagem, Niterói-RJ, 24210-346 Brazil

[c]Department of Chemistry – Ångström Laboratory, Uppsala University, Box 538, SE-751 21 Uppsala, Sweden

[*]Corresponding author, Email: matskg@kth.se

Supporting information

Synthesis:

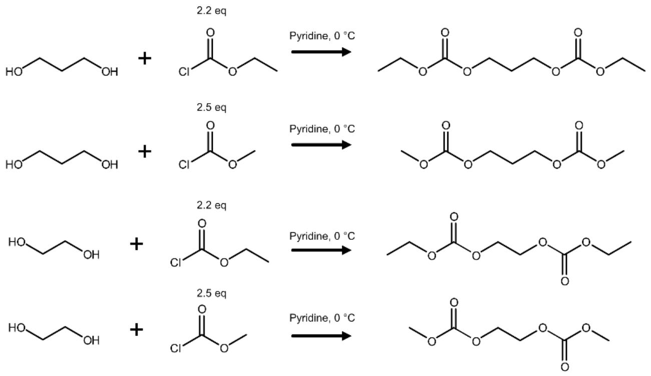

Table S1: Quantities of synthesis components

PE							
1,3-Propanediol		Ethyl chloroformate		Pyridine		Dimethylethanolamine	
g	mmol	g	mmol	g	mmol	g	mmol
5	65.7	15.7	144.6	22.9	289.1	6.0	67.3

PM							
1,3-Propanediol		Methyl chloroformate		Pyridine		Dimethylethanolamine	
g	mmol	g	mmol	g	mmol	g	mmol
5	65.7	15.5	164.3	26.0	328.6	6.8	76.5

EE							
1,2-Ethanediol		Ethyl chloroformate		Pyridine		Dimethylethanolamine	
g	mmol	g	mmol	g	mmol	G	mmol
5	78.0	18.6	171.7	27.2	343.4	7.1	80.0

EM							
1,2-Ethanediol		Methyl chloroformate		Pyridine		Dimethylethanolamine	
g	mmol	g	mmol	g	mmol	G	mmol
5	78.0	18.4	195.1	30.9	390.2	8.1	90.9

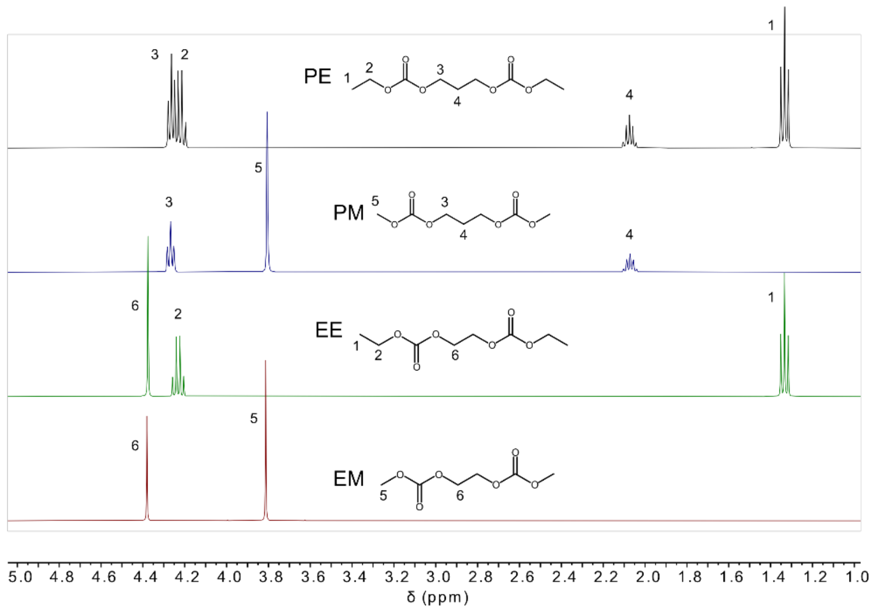

Figure S1: Summarized ^{1}H-NMR spectra of synthesized dicarbonates after purification and drying. (400 MHz, 298 K, $CDCl_3$)

^{1}H-NMR of PE ($CDCl_3$, 400 MHz, 298 K, δ/ppm): δ 4.27 (t, 4H, *J* = 6.2 Hz), δ 4.22 (q, 4H, *J* = 7.2 Hz), δ 2.07 (quint, 2H, *J* = 6.3 Hz), δ 1.33 (t, 6H, *J* = 7.1 Hz)

^{1}H-NMR of PM ($CDCl_3$, 400 MHz, 298 K, δ/ppm): δ 4.27 (t, 4H, *J* = 6.2 Hz), δ 3.80 (s, 6H), δ 2.07 (quint, 2H, *J* = 6.3 Hz)

^{1}H-NMR of EE ($CDCl_3$, 400 MHz, 298 K, δ/ppm): δ 4.38 (s, 4H), δ 4.23 (q, 4H, *J* = 7.2 Hz), δ 1.33 (t, 6H, *J* = 7.1 Hz)

^{1}H-NMR of EM ($CDCl_3$, 400 MHz, 298 K, δ/ppm): δ 4.38 (s, 4H), δ 3.80 (s, 6H)

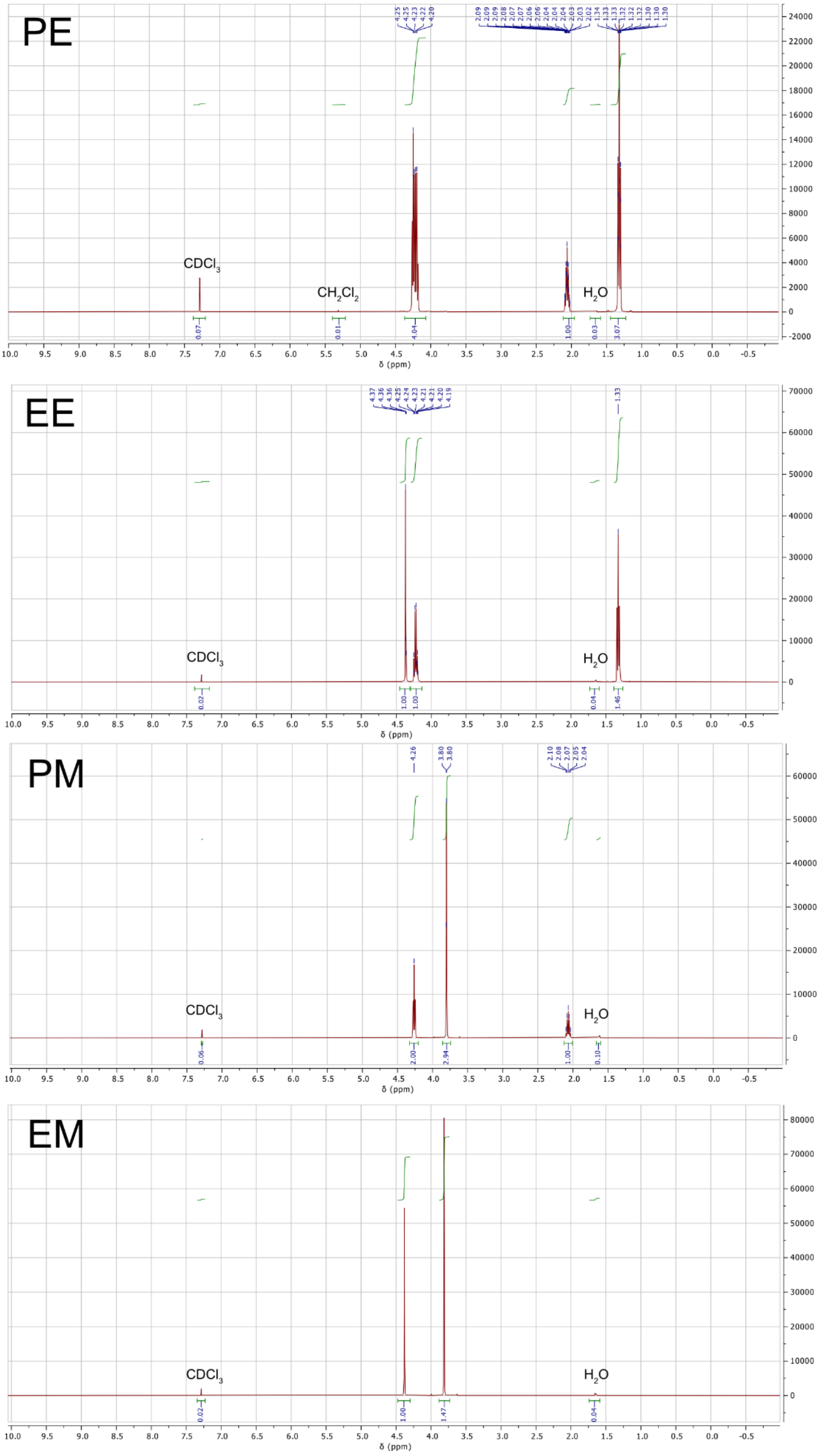

Figure S2: Full 1H-NMR spectra of PE, EE, PM and EM.

Physical properties:

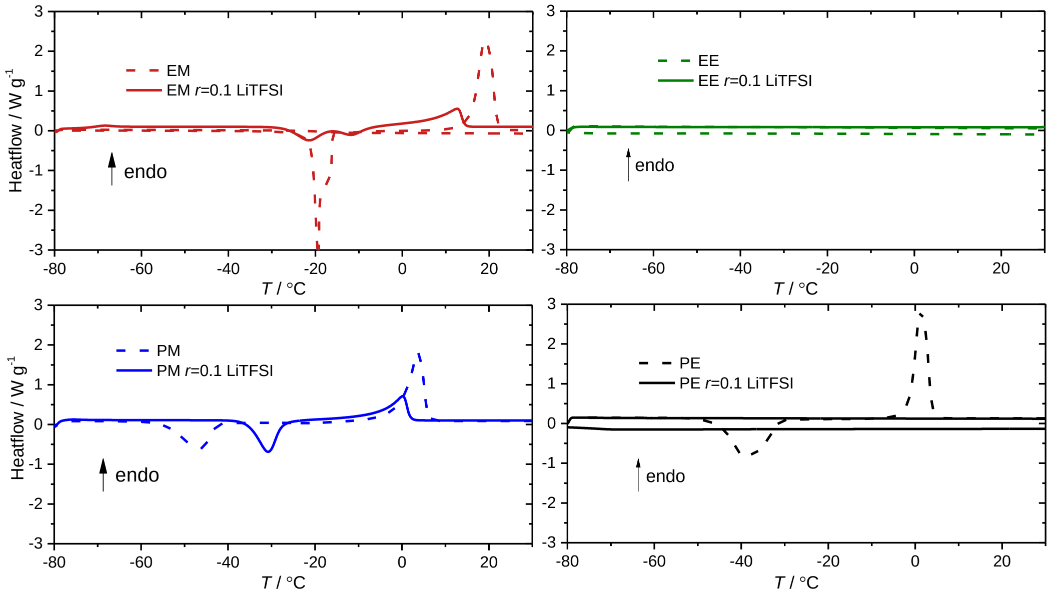

Figure S3: DSC thermograms for pure dicarbonates and with r =0.1 LiTFSI. The heating and cooling rate was set at 5 °C min^{-1}

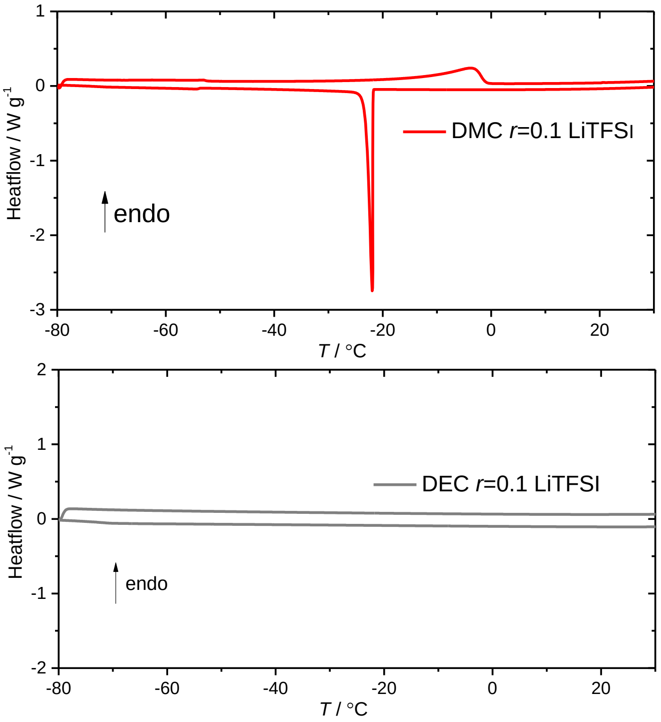

Figure S4: DSC thermograms for DMC and DEC with r = 0.1 LiTFSI. The heating and cooling rate was set at 5 °C min^{-1}

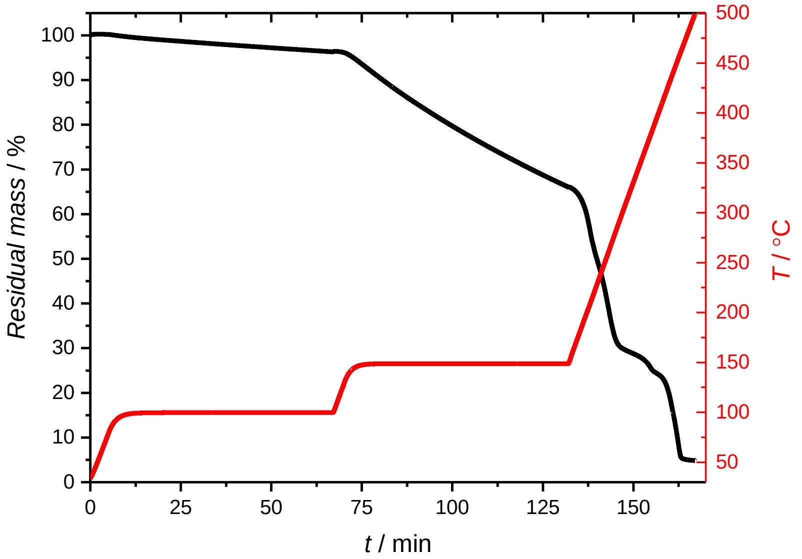

Figure S5: TGA thermogram of incremental temperature step program at 100 °C and 150 °C for 1 hour for PE with *r*=0.1 LiTFSI in an open cup.

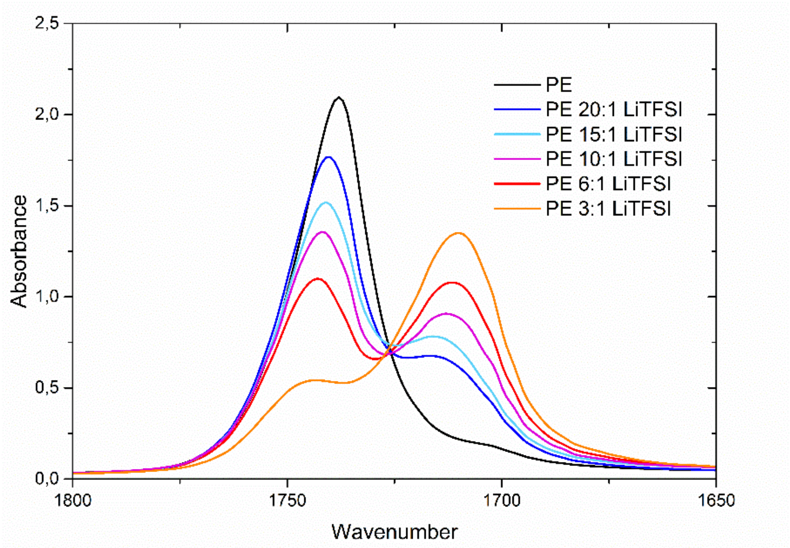

Figure S6: FTIR spectra of the carbonyl band of PE at different salt concentrations, showing a band at 1730 cm^{-1} representing uncoordinated C=O and a band at 1710 cm^{-} for coordinated C=O

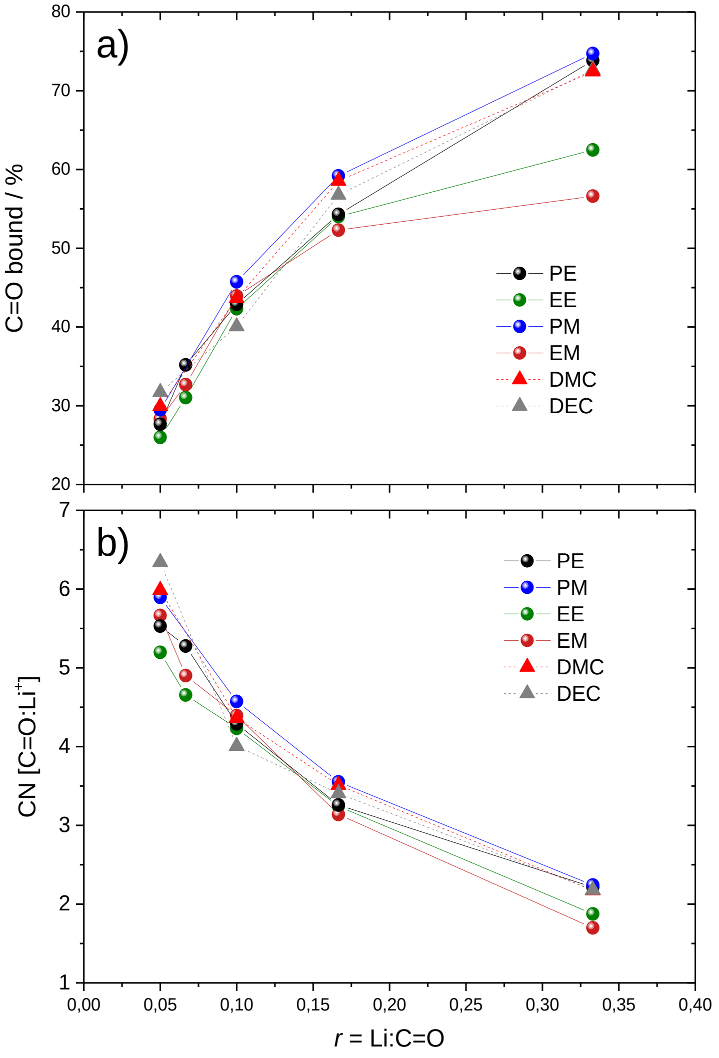

Figure S7: a) Proportion of bound carbonyls and b) carbonyl coordination number (CN) as a function of the lithium to carbonyl ratio (*r*) without taking the extinction factor ratio into account.

Calculating carbonyl coordination number:

To calculate the carbonyl CN, the following formula was used:

$$CN = \frac{A_C}{A_F + A_C} * \frac{n_{C=O}}{n_{Li^+}}$$

Where A_c and A_F are the areas of the coordinating and free carbonyl peaks. $n_{C=O}/n_{Li+}$ represents the ratio of carbonyl groups to lithium ions in the solution. This formula was used to find the CN in Figure S7

To take the different extinction coefficients into account, the extinction coefficient ratio was introduced into the formula:

$$CN = \frac{A_C}{\frac{\varepsilon_C}{\varepsilon_F} * A_F + A_C} * \frac{n_{C=O}}{n_{Li^+}}$$

$\frac{\varepsilon_C}{\varepsilon_F} = 1.508$ for DEC and $\frac{\varepsilon_C}{\varepsilon_F} = 1.391$ for DMC (Lim et. al.)[1]

Molecular Dynamics

Table S1 shows the composition used in the simulations, and the **figure S1** depicts the carbonate dimers and the LiTFSI salt. For the carbonate molecules, DMC and DEC, we used the same mol% as described here.

Table S2: Total and partial number of molecular components that comprise the systems studied.

C=O:Li+ (salt %)	PM	PE	EM	EE	Li^+	$TFSI^-$	Total
**6:1 (25 mol%)**	375	375	375	375	125	125	500
**10:1 (16 mol%)**	420	420	420	420	80	80	500
**20:1 (9 mol%)**	455	455	455	455	45	45	500

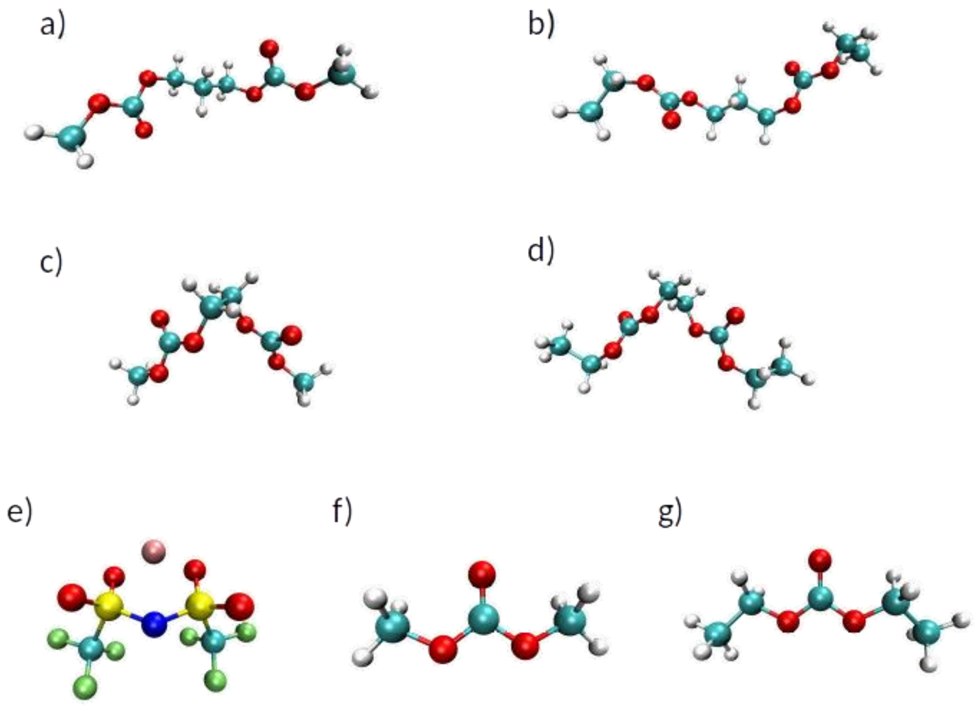

Figure S8: DFT-optimized dicarbonates (PM: a; PE: b; EM: c; and EE: d), LiTFSI, and the linear carbonates dimethyl carbonate (DMC), and diethyl carbonate (DEC) molecular structures used in this work. Notice that EM is the shortest LC, whereas the PE is the longest one. Coloring: grey, cyan, red, yellow, lime, and pink sphere colors stand for hydrogen, carbon, oxygen, sulfur, fluorine atoms, and lithium cation, respectively.

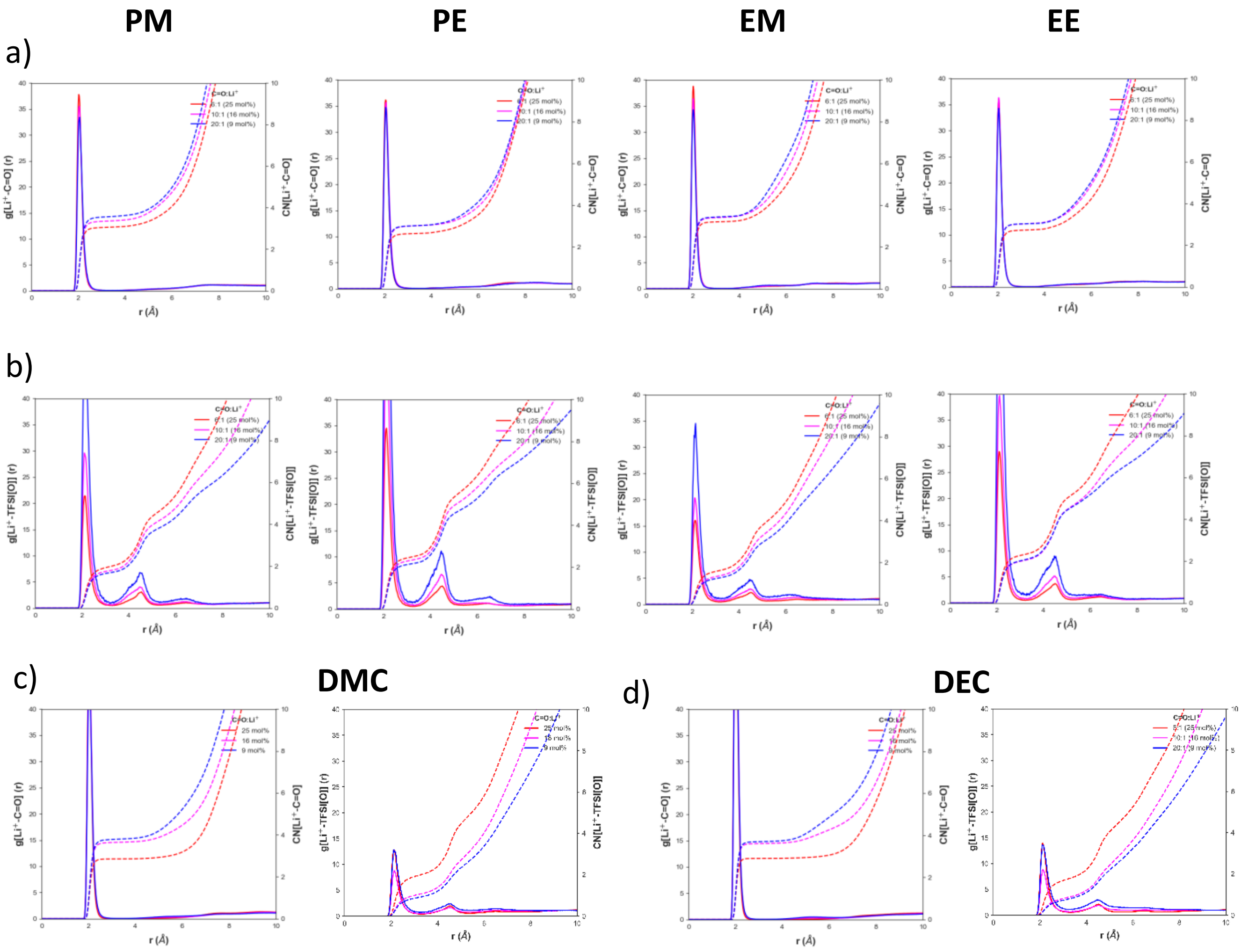

Figure S9: Radial distribution function (RDF) between Li cation and oxygen carbonate atoms of the dicarbonates (a), oxygen atoms of TFSI anion (b), oxygen carbonate and oxygen atoms of TFSI anion of DMC (c) and DEC (d). Dashed lines (to the right, vertical axis) are indicated of the coordination number of oxygen carbonates with respect to the Li-cation

The RDFs shown in **figure S9-a** do not present any difference for ethyl-ending dicarbonates (PE and EE), while for methyl-ending ones, their main peaks are slightly higher than the former ethyl-ending groups. For those methyl-ending groups, the difference in the main peak is visible between the highest salt concentration (25 mol%) and the lowest one (9 mol%) simulated.

For the Li^+-TFSI[O] pair distribution function in **figure S9-b**, ethyl-ending systems (PE and EE) show general higher intensities than methyl-ending ones for all concentrations. Also, it can be observed that the shorter the dimer length (PE > EE > PM > EM), the lower is the RDF intensity. For both analysis, Li^+-C=O and Li^+-TFSI, the positions of the main peaks do not change for all the systems.

For the linear carbonates (DMC and DEC in **figure S9-c** and **-d**), the main RDFs peak are very similar, as well as the coordination number (CN) between DMC and DEC counterparts. For the highest concentration, CN is about 2.9. For 16 and 9 mol%, 3.6 and 3.8, respectively. This result and the corresponding ones to the dimers are well discussed in the main text.

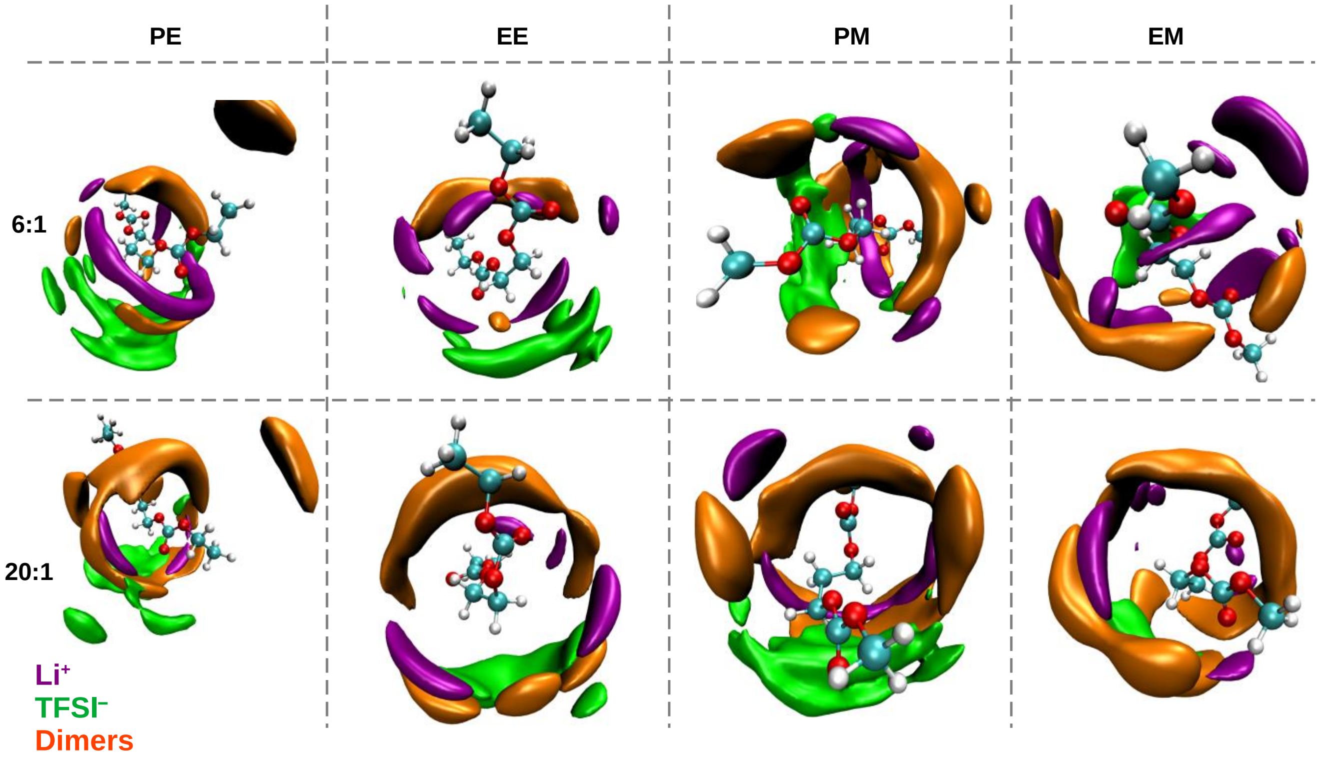

Figure S10: Spatial distribution functions (SDF) showing how each system's components are arranged, on average, along the trajectory. The isosurface colors are shown on bottom left. Each of the dicarbonates are shown explicitly. Two different salt concentration were analyzed and are disposed horizontally (6:1, 25 mol%, upper line; 20:1, 9 mol%, bottom line). SDFs were built up to a 10 Å range using 5000 frames extracted from the simulation trajectories.

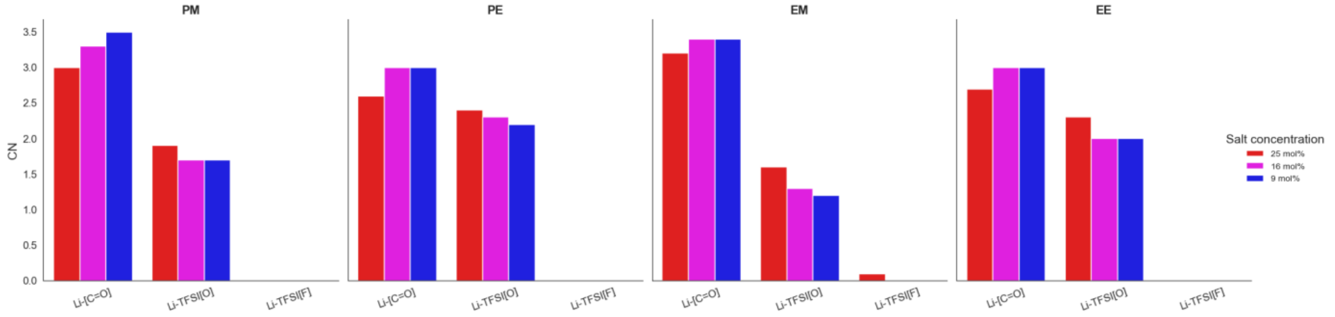

Figure S11: Coordination number (CN) for the atoms shown in the x-axis with respect to the Li cation for each salt concentration. They were calculated taking the RDF minima as the stopping criteria.

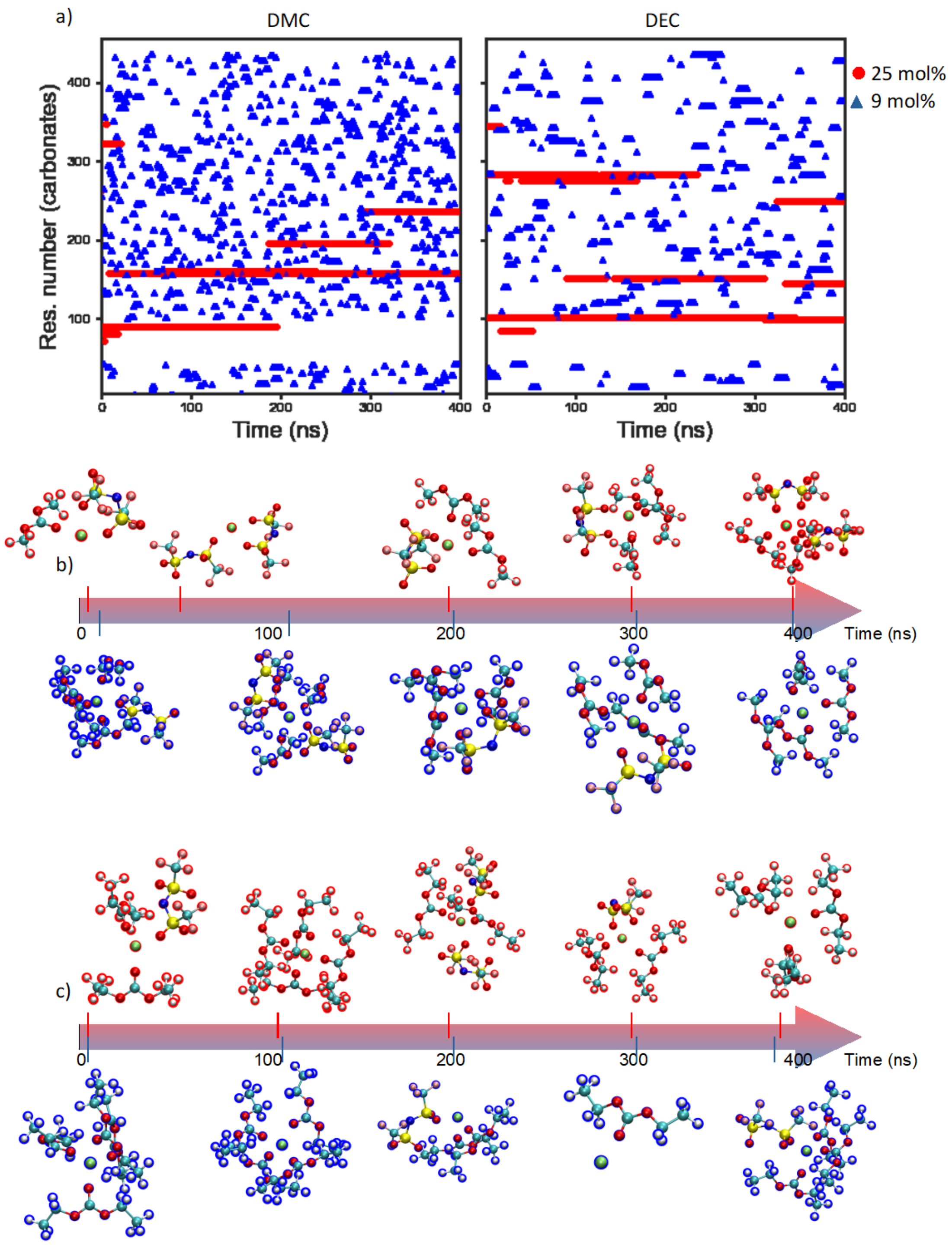

Figure S12: Time evolution plots (a) for a Li^+ in 'contact' to DMC and to DEC, which means it is a certain distance from the oxygen carbonyl. The plot for the whole simulation time (400 ns). A few representative snapshots along the simulation time for DMC (b) and DEC (c) representing how the interactions between the components of the systems are happening.

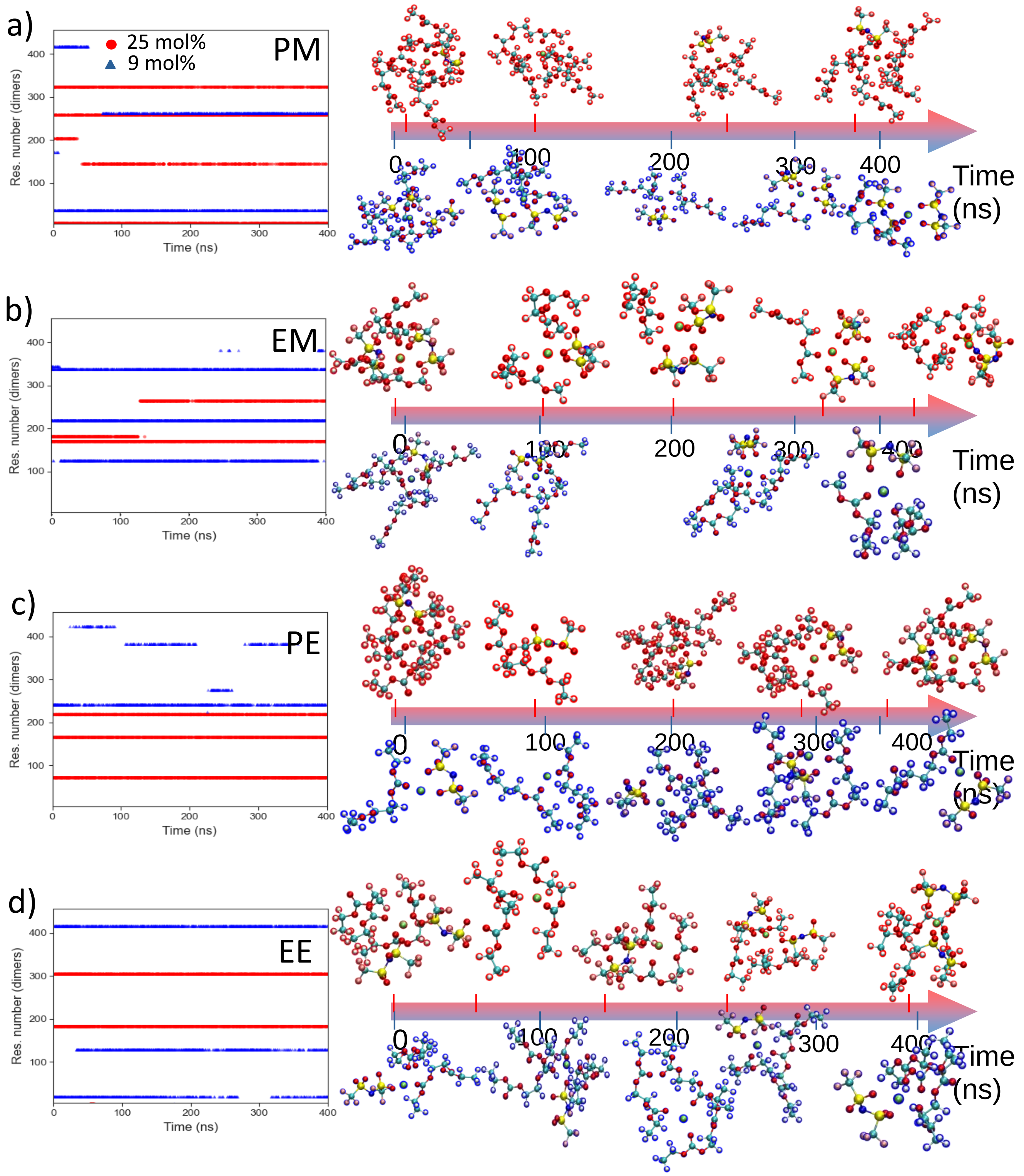

Figure S13: Time evolution plots for a Li+ in ‘contact’ to dicarbonates, which means if it is at a certain distance from the oxygen carbonyl the plot is dotted according to the dimer’s residue number. The plot is for the whole simulation time (400 ns).

References:

1. Lim, C.; Kim, J. H.; Chae, Y.; Lee, K.-K.; Kwak, K.; Cho, M., Solvation Structure around Li^+ Ions in Organic Carbonate Electrolytes: Spacer-Free Thin Cell IR Spectroscopy. *Analytical Chemistry* **2021,** *93* (37), 12594-12601.
